# Laser-Induced MXene-Functionalized Graphene Nanoarchitectonics-Based Microsupercapacitor for Health Monitoring Application

**DOI:** 10.1021/acsnano.3c07319

**Published:** 2023-10-04

**Authors:** Sujit Deshmukh, Kalyan Ghosh, Martin Pykal, Michal Otyepka, Martin Pumera

**Affiliations:** † Future Energy and Innovation Laboratory, Central European Institute of Technology, Brno University of Technology, Purkyňova 123, 61200 Brno, Czech Republic; ‡ Regional Centre of Advanced Technologies and Materials, Czech Advanced Technology and Research Institute (CATRIN), Palacký University in Olomouc, Šlechtitelů 27, 783 71 Olomouc, Czech Republic; § IT4Innovations, VŠB-Technical University Ostrava, 17. listopadu 2172/15, 708 00 Ostrava-Poruba, Czech Republic; ∥ Faculty of Electrical Engineering and Computer Science, VSB - Technical University of Ostrava, 17. listopadu 2172/15, 70800 Ostrava, Czech Republic; ⊥ Department of Chemical and Biomolecular Engineering, Yonsei University, 50 Yonsei-ro, Seodaemun-gu, Seoul 03722, Korea; # Department of Medical Research, China Medical University Hospital, China Medical University, No. 91 Hsueh-Shih Road, Taichung 40402, Taiwan

**Keywords:** Laser-induced MXene, laser-induced graphene, covalent bonding, microsupercapacitor, biomonitoring device

## Abstract

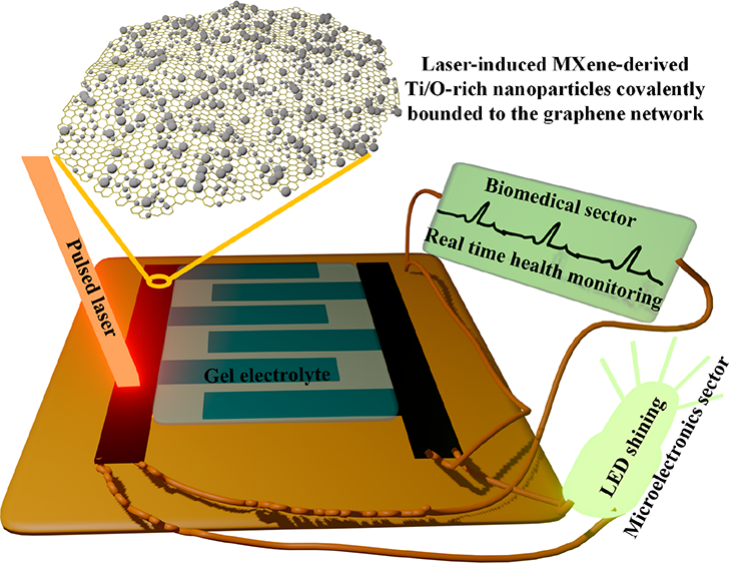

Microsupercapacitors (micro-SCs) with mechanical flexibility have the potential to complement or even replace microbatteries in the portable electronics sector, particularly for portable biomonitoring devices. The real-time biomonitoring of the human body’s physical status using lightweight, flexible, and wearable micro-SCs is important to consider, but the main limitation is, however, the low energy density of micro-SCs as compared to microbatteries. Here using a temporally and spatially controlled picosecond pulsed laser, we developed high-energy-density micro-SCs integrated with a force sensing device to monitor a human body’s radial artery pulses. The photochemically synthesized spherical laser-induced MXene (Ti_3_C_2_T_*x*_)-derived oxide nanoparticles uniformly attached to laser-induced graphene (LIG) act as active electrode materials for micro-SCs. The molecular dynamics simulations and detailed spectroscopic analysis reveal the synergistic interfacial interaction mechanism of Ti–O–C covalent bonding between MXene and LIG. The incorporation of MXene nanosheets improves the graphene sheet alignment and ion transport while minimizing self-restacking. Furthermore, the micro-SCs based on a nano-MXene-LIG hybrid demonstrate high mechanical flexibility, durability, ultrahigh energy density (21.16 × 10^–3^ mWh cm^–2^), and excellent capacitance (∼100 mF cm^–2^ @ 10 mV s^–1^) with long cycle life (91% retention after 10 000 cycles). Such a single-step roll-to-roll highly reproducible manufacturing technique using a picosecond pulsed laser to induce MXene-derived spherical oxide nanoparticles (size of quantum dots) attached uniformly to laser-induced graphene for biomedical device fabrication is expected to find a wide range of applications.

The booming of miniaturized portable and wearable electronics such as stretchable displays,^1^ force-sensitive detectors (FSDs),^2^ artificial electronics skin,^3^ and wearable microsensors^4,5^ have raised the demand of power sources (batteries and supercapacitors) that are capable of working in flexible deformation or to integrate with a variety of electronics devices. A key branch of such modern electronic sectors deals with health monitoring sensors that can collect real-time physiological and electrophysiological data from the human body.^6^ However, microbatteries are still the devices of choice for this type of application despite their slow charge/discharge processes and limited life cycle.^7^ Microsupercapacitors (micro-SCs), especially with planar interdigitated structures, are promising alternatives to microbatteries due to their high power densities, longer lifetimes, and much faster charge/discharge rates.^8,9^ The main challenge in using micro-SC devices in modern electronics sectors is to increase the energy density to a level comparable to or even exceeding those of microbatteries without compromising the electrochemical properties.

A general strategy to improve the energy density of SCs is to create porous conductive electrode materials with an adequate high packing density to maximize the utilization of the small size of micro-SCs. In this context, graphene sheets are ideal candidates because of their ultrahigh surface area (2630 m^2^ g^–1^), excellent electrical conductivity, and rich surface chemistry.^10−12^ To commercialize graphene, methodologies have been developed for producing graphene on a large scale and roll-to-roll compatible thin films without compromising the fundamental properties of graphene sheets.^13,14^ Laser-induced photothermal conversion of a nonconductive carbon source into a conductive graphene structure has recently emerged as a roll-to-roll compatible technique for producing laser-induced graphene (LIG).^15^ This approach allows graphene to assemble into many intriguing structures such as one-dimensional fibers^16,17^ and three-dimensional foams^18^ as well as the production of desired patterns or geometries by adjusting laser settings.^19,20^ Although LIG-based micro-SCs have large gravimetric capacitances, they are constrained by a poor volumetric performance. A probable explanation for this behavior is the strong intersheet π–π interaction, which, although boosting the packing density, does not allow for high ion accessibility. The pioneering concept of laser processing graphene by El-Kady et al. for micro-SCs has delivered outstanding power output but fails to achieve the high areal capacitance (<5 mF cm^–2^) due to the stacking problem.^21^ Because pure electrical double layers of graphene have limited capacitance, the stacking problem is strategically mitigated by combining LIG with highly electroactive materials and pseudocapacitive materials that have larger capacitance.^22,23^

A new class of graphene-analogous materials, 2D transition-metal carbides, and nitrides (known as MXene) have gained huge interest from researchers due to their 2D structure and customizable surface chemistry, which provide MXene with a plethora of exciting features such as ultrahigh metallic conductivity (15 100 S cm^–1^), strong hydrophilicity, and notable mechanical capabilities.^24,25^ As a result, MXene has shown enormous promise in energy storage applications, such as SCs as well as lithium- and sodium-ion batteries.^26−29^ Since the first discovery of 2D MXene (Ti_3_C_2_T_*x*_) in 2011 by Gogotsi and colleagues, it has been the most used transition-metal carbide (TMC) for energy storage applications.^30^ However, like other 2D materials, the inevitable agglomeration and layer-by-layer restacking due to the high van der Waals force severely reduce the electrochemically active sites and limit the permeability of electrolyte ions.^31^ As a result, the dense MXene sheets suffer from low specific capacitance (100–300 F g^–1^) and poor cyclic stability, which need to improve further.^28,32^ Thus, the intercalation of MXene nanosheets between the 3D network of LIG would be a very effective approach to inhibiting the self-restacking of both graphene and MXene flakes. However, the process to fabricate MXenes in the form of ultrathin flakes/particles with a few-nanometer thickness/diameter that can intercalate between the graphene network is a key challenge to overcome. Beyond the production of nanosized MXene, the ease with which they may be integrated into devices represents another key challenge in achieving the potential of MXene for applications.

Herein we reported a versatile process to fabricate nano-MXene (Ti_3_C_2_T_*x*_) functionalized and cross-linked with graphene platelets through Ti–O–C covalent bonding. The approach involves a single-step lasing process on an MXene-coated polyimide (PI) sheet by a diode-pumped Nd:YAG solid pulsed laser beam. When ablated by an Nd:YAG solid pulsed laser, the delaminated MXene sheet absorbs the IR energy and generates high temperature (over 2000 K) on the PI sheet within a rapid uptake time (submillisecond time scale) which causes the self-assembly and ordering of the C–C bond to form an MXene-decorated 3D graphene nanostructure.^33^ It is known that Ti_3_C_2_T_*x*_, for example, degrades at roughly 200 °C owing to surface group collapse, whereas Ti_3_C_2_ is projected to endure up to 1000 °C.^33,34^ Hence, laser irradiation first creates Ti–O–C bonding and π–π bridging at the MXene graphene interface, and then, it decays the Ti_3_C_2_T_*x*_ sheets into Ti-rich oxide nanoparticles by thermal oxidation that are decorated over the 3D network of LIG. However, the MXene-functionalized LIG was hydrophobic in nature; therefore, we used a room-temperature oxygen plasma treatment to modify the underlying surface wettability of MXene-functionalized LIG, allowing better electrode–electrolyte interaction. Even the laser-processed MXene has now emerged as a promising material for optoelectronics, sensors, or microsupercapacitors.^35−37^ The proposed approach is easily scalable, and devices are prepared on a wide scale while maintaining flexibility. All of the devices developed demonstrated energy densities equivalent to microbatteries while retaining good rate performance, cycle stability, and mechanical flexibility. Direct patterning of laser-induced nano-MXene intercalated graphene would enable roll-to-roll manufacturing of active electrode material for a host of applications from energy storage devices to biomonitoring.

## Results and Discussion

### Synthesis and Characterization of MXene-Derived Oxide-Particle-Functionalized Graphene Sheets

**Figure 1:**
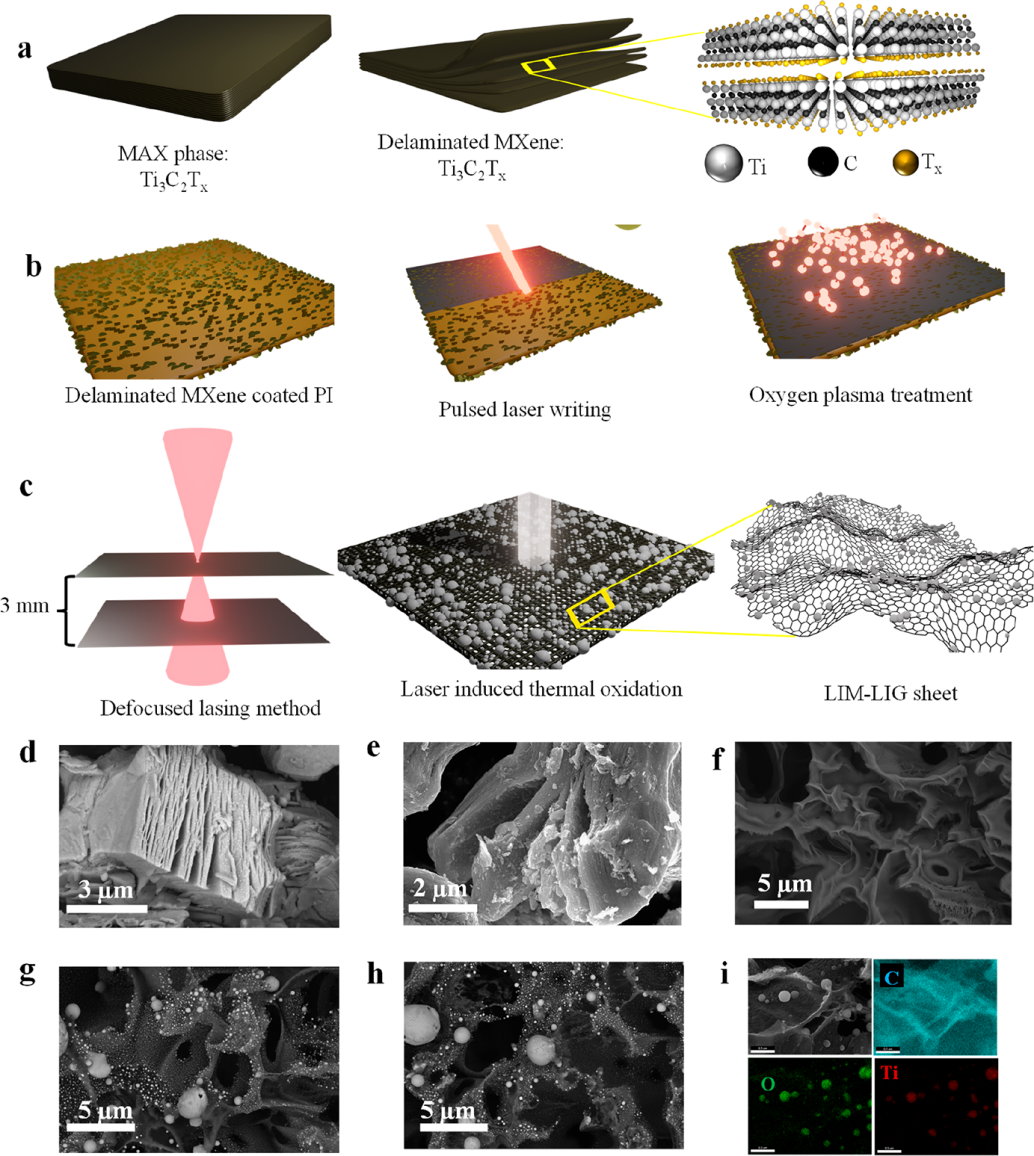
Scheme for the synthesis of the O-LIM-LIG hybrid using picosecond pulsed laser and morphological characterization. (a) Stacked Ti_3_C_2_T_*x*_ MXene and delaminated Ti_3_C_2_T_*x*_ MXene with increased interlayer spacing. Schematic model of Ti_3_C_2_T_*x*_ MXene layers composed of Ti, C, and T_*x*_ atoms. (b) Scheme of the microfabrication steps of the O-LIM-LIG hybrid. (c) Thermal oxidation of delaminated MXene due to appreciable IR energy absorption results in spherical LIM particles attached to LIG, as demonstrated by a defocused lasing mechanism. SEM top view of (d) stacked Ti_3_C_2_T_*x*_ MXene, (e) delaminated Ti_3_C_2_T_*x*_ MXene, (f) LIG, (g) LIM-LIG, and (h) the O-LIM-LIG hybrid showing the uniform distribution of spherical LIM across the LIG surface. (i) Elemental mapping images for the O-LIM-LIG revealing the presence of Ti, O, and C. Scale bar 0.5 μm.

The schematic diagram to prepare the MXene-decorated LIG is shown in Figure 1a,b. First, the delaminated MXene (Ti_3_C_2_T_*x*_) was spin-coated (500 rpm; 60 s) on the flexible PI sheet followed by pulsed laser (Nd:YAG) writing on the MXene-coated PI sheet. Note that we have used here the defocused method (Figure 1c), resulting in multiple lases in a single run of the pulsed laser. This method allows us to simply adjust the laser’s spot size while maintaining consistent dot density. Lowering the substrate by ∼3 mm below the focal point increases the spot size resulting in multiple lasing on a given spot while maintaining the density of the laser spot constant. Because each spot may lase many times in a single laser pass, this defocused approach enhances processing speed. The sheet resistance of defocused LIG (∼20 Ω sq^–1^) is also lower as compared to LIG prepared at the focal length. In this process, an in situ decoration/bonding of the laser-induced MXene derive Ti-rich oxide nanoparticles (LIM) inside the 3D porous network of graphene was achieved. The laser process mechanism is further elaborated in the following discussion.

When exposed to an IR laser pulse (Figure 1c), the MXene-coated PI sheet absorbs the IR energy and develops a high local temperature (over 2000 K) within a rapid uptake time (submillisecond).^33^ This rapid initial uptake leads to the formation of carbonized steam (O_2_, CO, CO_2_, CH_4_, N_2_) from the PI sheet resulting in a 3D porous interconnected structure of LIG (Figure 1f). On the other hand, the carbon source from the MXene explosively vaporizes, and metal ion oxidation takes place when the delaminated MXene nanosheets with abundant oxygen-containing functional groups absorb the IR energy.^33^ This instantaneous high-temperature thermal oxidation therefore degrades the Ti_3_C_2_T_*x*_ sheets into spherical Ti-rich oxide nanoparticles. However, before being employed as an SC electrode, it was activated further by utilizing oxygen (O_2_) plasma treatment. A comparative analysis of surface morphologies for the pristine MXene, delaminated MXene, LIG, and laser-processed MXene-graphene samples are discussed next.

The successful delamination of Ti_3_C_2_T_*x*_ MXene and LIM-decorated graphene electrodes was confirmed by scanning electron microscopy (SEM). The surface morphology of the pristine Ti_3_C_2_T_*x*_ MXene is displayed in Figure 1d, confirming the multilayer stack of Ti_3_C_2_T_*x*_ with an accordion-like structure. After delamination with a strong oxidizing agent (viz. DMSO), the surface oxidation of Ti_3_C_2_ leads the surface to become rougher (Figure 1e). Figure 1g illustrates the uniform distribution of spherical LIM on the LIG network (called: LIM-LIG), where the LIM particle size ranges from 1 μm to the subnanometer range. The surface morphology of O_2_ plasma-treated LIM-LIG (called: O-LIM-LIG) is similar to that of LIM-LIG (Figure 1h). The heterostructure with this sort of distribution of LIM over graphene not only is helpful for improved space utilization but also efficiently prevents graphene sheets from self-restacking. Subsequently, in order to corroborate the uniform distribution of titanium-rich spherical particles across the LIG network, an additional elemental mapping analysis is carried out.

Elemental mapping images of the O-LIM-LIG (Figure 1i) and MXene (Supplementary Figure 1) were acquired by energy-dispersive X-ray spectroscopy (EDX) to see the surface element and to verify the surface decoration of LIM over the LIG network. These reveal the presence of C, O, and Ti in the O-LIM-LIG hybrid, which is further verified by Raman, XRD, and XPS analysis. The cross-sectional SEM images (Supplementary Figure 2) reveal that the O-LIM-LIG has a thickness of ∼107 μm, which was taken into account to compute the volumetric capacitance of the micro-SC devices.

**Figure 2:**
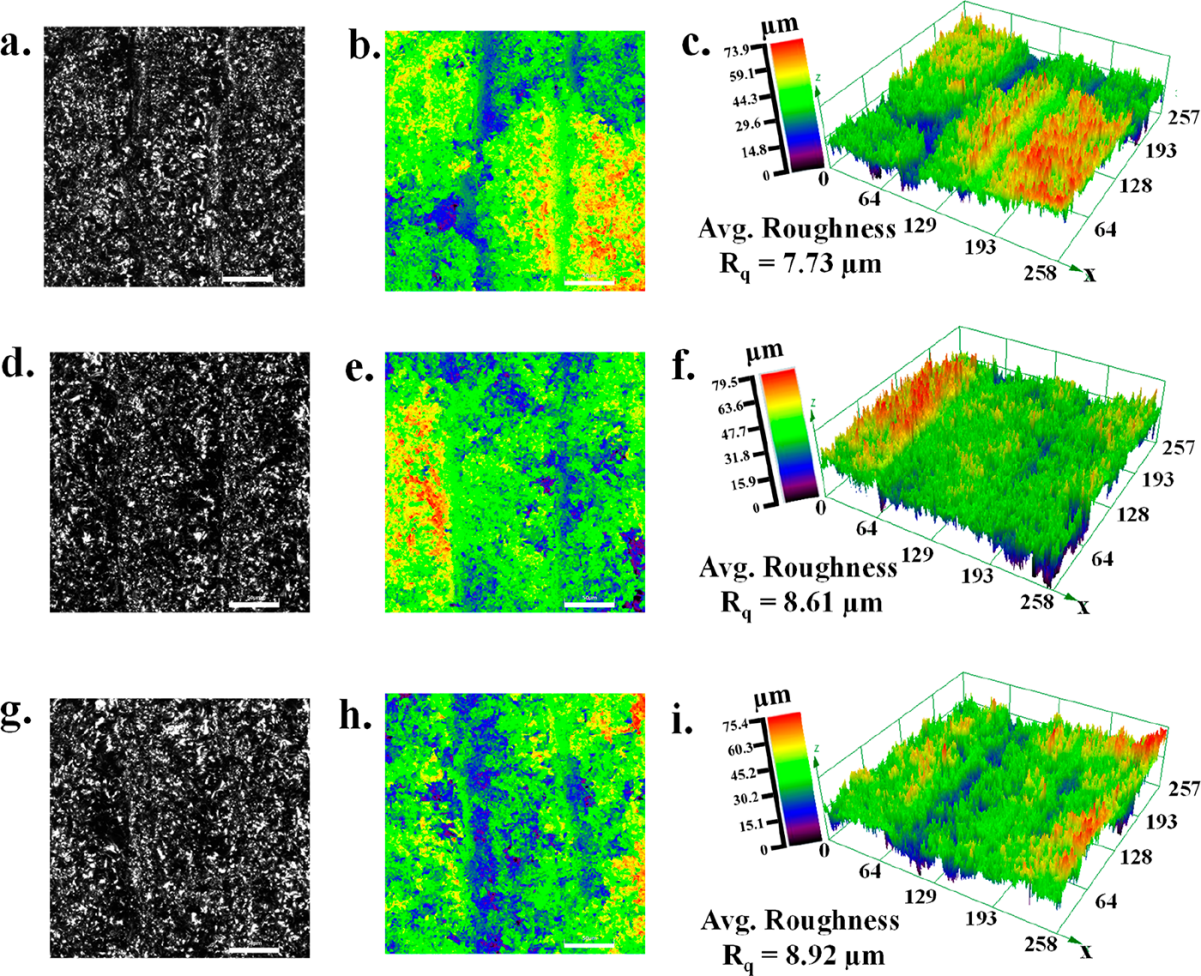
Confocal laser scanning microscope optical image and corresponding 2D and 3D false color profile of (a–c) LIG, (d–f) LIM-LIG, and (g–i) O-LIM-LIG. Scale bar ∼50 μm.

**Figure 3:**
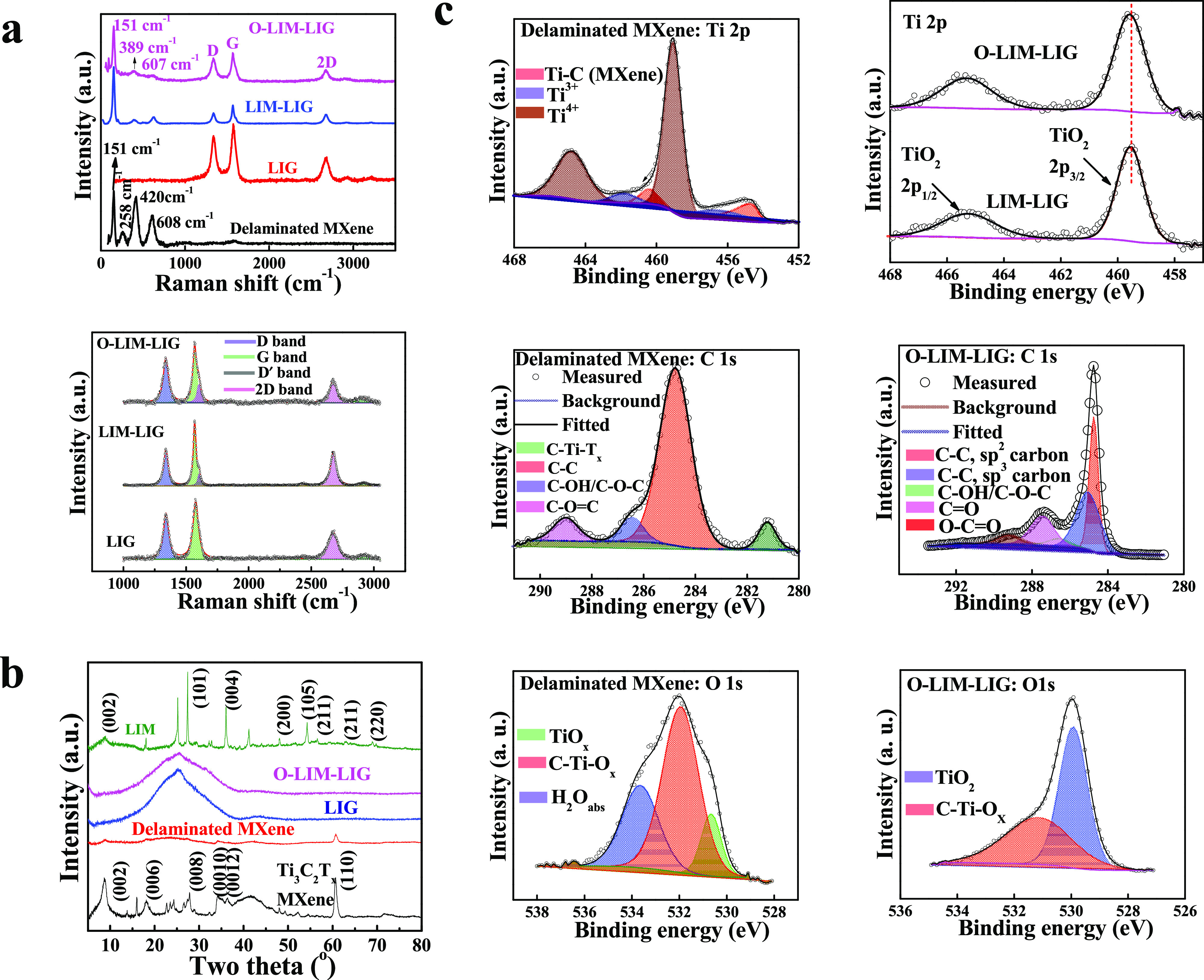
Physical characterizations. (a) Raman spectra of delaminated Ti_3_C_2_T_*x*_ MXene, LIG, LIM-LIG, and O-LIM-LIG films. Fitted Raman spectra were within the range of 1000–3000 cm^–1^. (b) XRD spectra of Ti_3_C_2_T_*x*_ MXene, delaminated Ti_3_C_2_T_*x*_ MXene, LIG, O-LIM-LIG, and LIM films. (c) Ti 2p, C 1s, and O 1s high-resolution XPS spectra of delaminated Ti_3_C_2_T_*x*_ MXene and O-LIM-LIG.

Figure 2 displays the optical images of LIG, LIM-LIG, and O-LIM-LIG captured by a confocal laser scanning microscope (CLSM). The topography (Figure 2a,d,g) is represented together with equivalent 2D (Figure 2b,e,h) and 3D (Figure 2c,f,i) false color image maps where distinct colors correspond to the various height profiles of the samples. The height profiling was used to estimate the average surface roughness (*R*_q_) of the samples. The *R*_q_ values of LIG, LIM-LIG, and O-LIM-LIG were calculated as 7.7, 8.6, and 8.9 μm, respectively. The O-LIM-LIG surface becomes rougher with LIM insertion into the LIG surface and subsequent treatment with the O_2_ plasma treatment. Note that the surface wettability is highly vulnerable to surface roughness,^38^ and increasing surface roughness leads to increased ion-accessible surface area, which is one of the fundamental factors for improving electrochemical SC performance.

Raman measurements were performed to determine the sorts of defects and growing disorders in the graphene network caused by the incorporation of MXene and the O_2_ plasma treatment. As displayed in Figure 3a, the delaminated MXene exhibits bands around ∼258, ∼420, and ∼608 cm^–1^ corresponding to the in-plane Ti–C vibration (E_g_ symmetry of Ti_3_C_2_), in-plane vibration of O atoms (E_g_ symmetry) of hydrogen-terminated MXene Ti_3_C_2_(OH)_2_, and mixed contribution of out-of-plane Ti–C vibration (A_1g_ symmetry of Ti_3_C_2_) and vibration of H atoms of Ti_3_C_2_(OH)_2_.^39,40^ The band at ∼151 cm^–1^ is the E_g_ vibrational mode of anatase TiO_2_ formed due to the spontaneous oxidation of Ti atoms.^41^ In the case of LIG’s Raman signal, three prominent peaks are visible (D ∼1340 cm^–1^, G ∼1574 cm^–1^, and 2D ∼2673 cm^–1^), which is consistent with the previous report.^9^ Interesting to note that after the laser process and oxidization by plasma treatment, the O-LIM-LIG exhibits three peaks at ∼151, ∼389, and ∼607 cm^–1^ in addition to D, G, and 2D peaks. These are the characteristic Raman active modes of anatase TiO_2_ particles with symmetries E_g__(1)_, B_1g__(1)_, A_1g,_ and E_g(3)._ The Raman results confirmed the formation of anatase TiO_2_ phase from on the Ti_3_C_2_T_*x*_ surface during the lasing process.

To further clarify the types of defects due to the formation of anatase Ti-rich oxide particles inside the LIG network, Raman spectra of LIG, LIM-LIG, and O-LIM-LIG are further fitted with the Lorentzian function (Figure 3a), and the extracted fitted parameters are listed in the Materials and Methods section. The single Lorentzian peak fitting of LIG’s 2D band with a full width at half-maximum of 86 cm^–1^ confirms the presence of a few layers of graphene stacked along the *c* axis. Note that three significant changes are visible when comparing the Raman spectra of LIG with LIM-LIG and O-LIM-LIG; 2D band blueshift of ∼3 cm^–1^, increment of the *I*_D_/*I*_G_ ratio, and the presence of an asymmetric G peak or appearance of an additional D′ peak at ∼1605 cm^–1^. The 2D band blueshift is caused by bond angle disorder and compressive stress at the LIG/MXene-derived oxide interfaces.^9^ The *I*_D_/*I*_G_ ratio is maximum for O-LIM-LIG, indicating the MXene-derived anatase TiO_2_ and oxygen functional groups have a strong impact on the in-plane sp^2^ domain of LIG. Finally, the I_D_/I_D′_ ratio (I_D_/I_D′LIM-LIG_ ∼5.5, I_D_/I_D′ O-LIM-LIG_ ∼3.8) indicates the defects linked with vacancies (Supplementary Table 1).^42^

X-ray diffraction (XRD) studies were performed (Figure 3b) to further validate the laser-induced transition of Ti_3_C_2_T_*x*_ to MXene-derived anatase TiO_2_ particles. The identified XRD peaks of delaminated Ti_3_C_2_T_*x*_ MXene and LIG are consistent with the previous published reports.^9,43^ However, the characteristic XRD peaks of MXene are not visible in the spectral analysis of O-LIM-LIG. Consider that delaminated MXene sheets undergo significant stratification and fragmentation by absorbing the laser energy, resulting in losing their 2D planar (002) stuck format and converting to Ti-rich oxide nanoparticles (as can be seen in Figure 1). However, it is worth noting that the transformation of Ti_3_C_2_T_*x*_ to anatase MXene-derived TiO_2_ appears to be ambiguous based on these XRD results. To clarify this ambiguity, we conducted a controlled experiment in which a glass slide was coated with delaminated MXene and treated with a laser thereafter. It is interesting to note that the residual presence of Ti_3_C_2_T_*x*_ still existed in LIM but shifted toward a high angle (∼8.9°). Meanwhile, a couple of additional XRD peaks emerged corresponding to the (101) and (004) planes of anatase TiO_2_. Hence, the dual presence of both MXene and MXene-derived anatase TiO_2_ nanoparticles is confirmed in the LIM samples.

As evidenced from XRD and Raman, the surface composition of the O-LIM-LIG has altered significantly after the laser writing and oxidation due to plasma treatment. To further confirm the surface chemical state/composition, X-ray photoelectron spectroscopy (XPS) characterization was carried out. The survey spectra report (Supplementary Figure 3) indicates the presence of Ti, C, and O in the three samples. An additional F peak is evident in the pristine MXene film due to residual F^–^ ions of the hydrofluoric solution used to etch the MAX phase. The atomic ratio of carbon to oxygen (C/O) is computed from the survey spectra, and we found that the C/O value is considerably reduced to 3.6 for the O-LIM-LIG compared to the LIG (C/O = 9.15). This implies that a significant number of oxygen functional groups were bound randomly either with planar sp^2^ hybridized benzene rings or with Ti atoms, which led to the higher sheet resistance of O-LIM-LIG as compared to other samples (see Supplementary Figure 8). The deconvolution of C 1s, O 1s, and Ti 2p clarifies it further (Figure 3C, Supplementary Figures 4 and 5). The binding energy values of the fitted peaks are listed in Supplementary Table 2. As illustrated in Figure 3C, several peaks are identified in the Ti 2p XPS spectrum of delaminated MXene, which is consistent with the previous findings.^43,44^ For LIM-LIG and O-LIM-LIG films, only TiO_2_ binding energy peaks are evident, whereas the Ti–C binding energy peak is absent. The C 1s XPS spectrum of MXene consists of four obvious peaks designated as C–C, C–OH, C–O=C, and C–Ti–T_*x*_ respectively. After laser treatment, the peak attributed to C–Ti−T_*x*_ disappeared for LIM-LIG and O-LIM-LIG. Furthermore, as shown in the O 1s spectrum, the intensity of the C–Ti–O_*x*_ peak is decreased for O-LIM-LIG than the Ti_3_C_2_T_*x*_, while a pronounced TiO_2_ peak is noted.

To reinforce this argument, we conducted a comparison of the Ti 2p XPS spectra (Supplementary Figure 6) for MXene, delaminated MXene, and LIM powder. In the case of MXene, distinct peaks corresponding to Ti–C (∼455 eV) and TiO_2_ (∼459 eV) were observed. Conversely, in the XPS spectrum of delaminated MXene, there was a decrease in the intensity of the Ti–C peak and an increase in the intensity of the TiO_2_ peak. This suggests that the oxidation level of the Ti atoms has increased during the solution-based delamination process. Intriguingly, the LIM sample did not exhibit a Ti–C peak; only peaks corresponding to the binding energy of TiO_2_ were evident. These results confirm the conversion of the Ti_3_C_2_T_*x*_ surface into MXene-derived TiO_2_ nanoparticles, which is in agreement with the Raman measurements.

The conversion of Ti_3_C_2_T_*x*_ into Ti-rich oxide nanoparticles and the induced oxygenated groups via plasma treatment have a significant influence on the underlying wettability of the O-LIM-LIG hybrid. Supplementary Figure 7 displays the water contact angle (WCA) values of LIG (WCA_LIG_ ∼110°) and LIM-LIG (WCA_LIM-LIG_ ∼94°), indicating that both are hydrophobic in nature, while the O-LIM-LIG electrode becomes superhydrophilic (WCA_O-LIM-LIG_ ∼0°). It could be correlated with the increased proportion of polar bonds C–O, C=O, and other oxygenated groups,^17^ which make the graphene edges more favorable to interact with water molecules, and eventually, the droplet sinks into the porous network of O-LIM-LIG. Increased hydrophilicity leads to increased ion-accessible surface area, which is critical for micro-SC device performance. However, the sheet resistance is compromised for the O-LIM-LIG (see Supplementary Figure 8) as compared to other electrodes due to the presence of oxygenated groups.

### Molecular Dynamics Simulation

**Figure 4:**
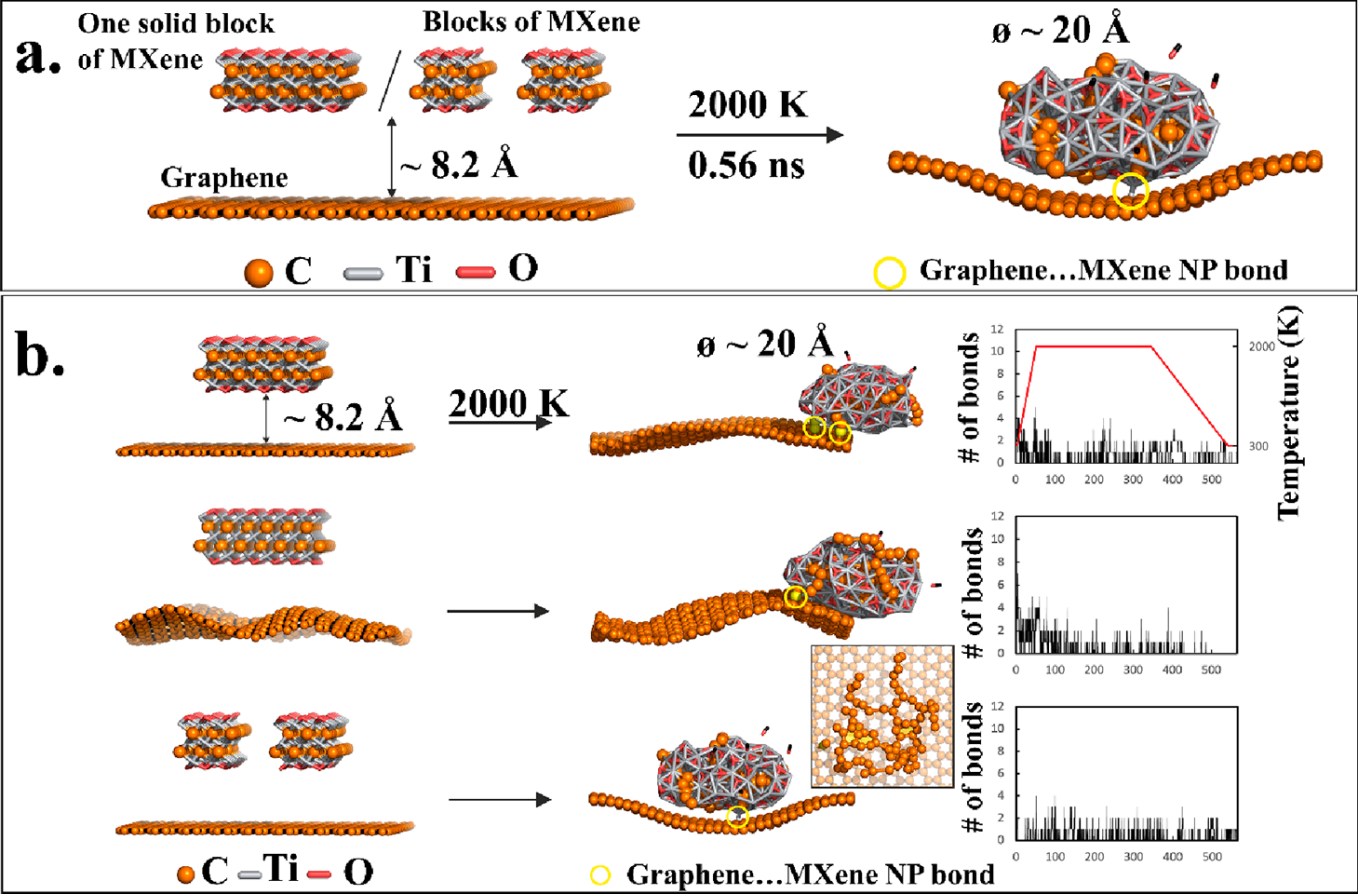
Interfacial interaction mechanism between MXene and LIG. (a) Initial and final snapshots taken from molecular dynamics simulation executed with reactive force field (ReaxFF) showing the formation of an MXene-derived nanoparticle covalently bound to the graphene surface (the covalent bonds are highlighted by yellow circles) at the high temperature (2000 K) induced by the laser pulse. (b) Snapshots taken from various MD simulations with different initial structures using ReaxFF at 2000 K showing the formation of covalently bonded nanoparticles on periodic graphene surfaces. The inset shows a detail of the interlaced carbon network of polyaromatic hydrocarbon-like structures with aliphatic side chains (Ti and O atoms are omitted for clarity). Aromatic cycles are colored in yellow. Graphs showing the number of direct covalent bonds between the MXene nanoparticle and the graphene surface (the average value is calculated from the last 50 ps). Colors: C (orange), Ti (gray), and O (red).

The formation of MXene-originated nanoparticles on LIG was studied by molecular dynamics (MD) simulations with a reactive force field ReaxFF (see Materials and Methods for details).^45^ We adopted the developed ReaxFF force field parameters, which were successfully used to investigate the dynamics and structural changes of heterostructures involving Ti_3_C_2_ MXenes.^46^ We focused on formation, morphological changes, and interaction of MXene nanoparticles with the graphene surface with variable flexibility (fixed/artificially wrinkled graphene) at elevated temperatures (2000 K) induced by the laser pulse. Regardless of the input structure, spherical oval-shaped particles were formed, some CO molecules were released, and carbon atoms constituted structures resembling functionalized polyaromatic hydrocarbons. The nanoparticles composed of Ti and O atoms, which were interlaced with polycyclic aromatic (hydro)carbon-like molecules with aliphatic carbon side chains (inset of Figure 4b, Ti and O atoms are omitted for clarity). The formed nanoparticles conjugated via covalent bonds with the graphene, which displayed a tendency to wrap the nanoparticle enlarging the contact surface (Figure 4 and Supplementary Figure 9) of both systems. The results of MD simulations support the experimental observations indicating the tendency of MXene to form Ti/O-rich nanoparticles covalently bound to graphene.

### Flexible Energy Storage Devices

The introduction of nano-MXene into the 3D network of LIG results in a substantially enhanced porosity and roughness in the LIM-LIG film as compared to LIG (Figures 1 and 2). Further O_2_ plasma treatment made LIM-LIG film superhydrophilic, which is beneficial for electrolyte ion accessibility inside the porous structure. Due to the distinctive and uniform decoration of nano-MXene/MXene-derived oxide (MDO) across the porous LIG, with the added benefit of superhydrophilicity, O-LIM-LIG was expected to serve as an innovative class of electrode material for SC application.

**Figure 5:**
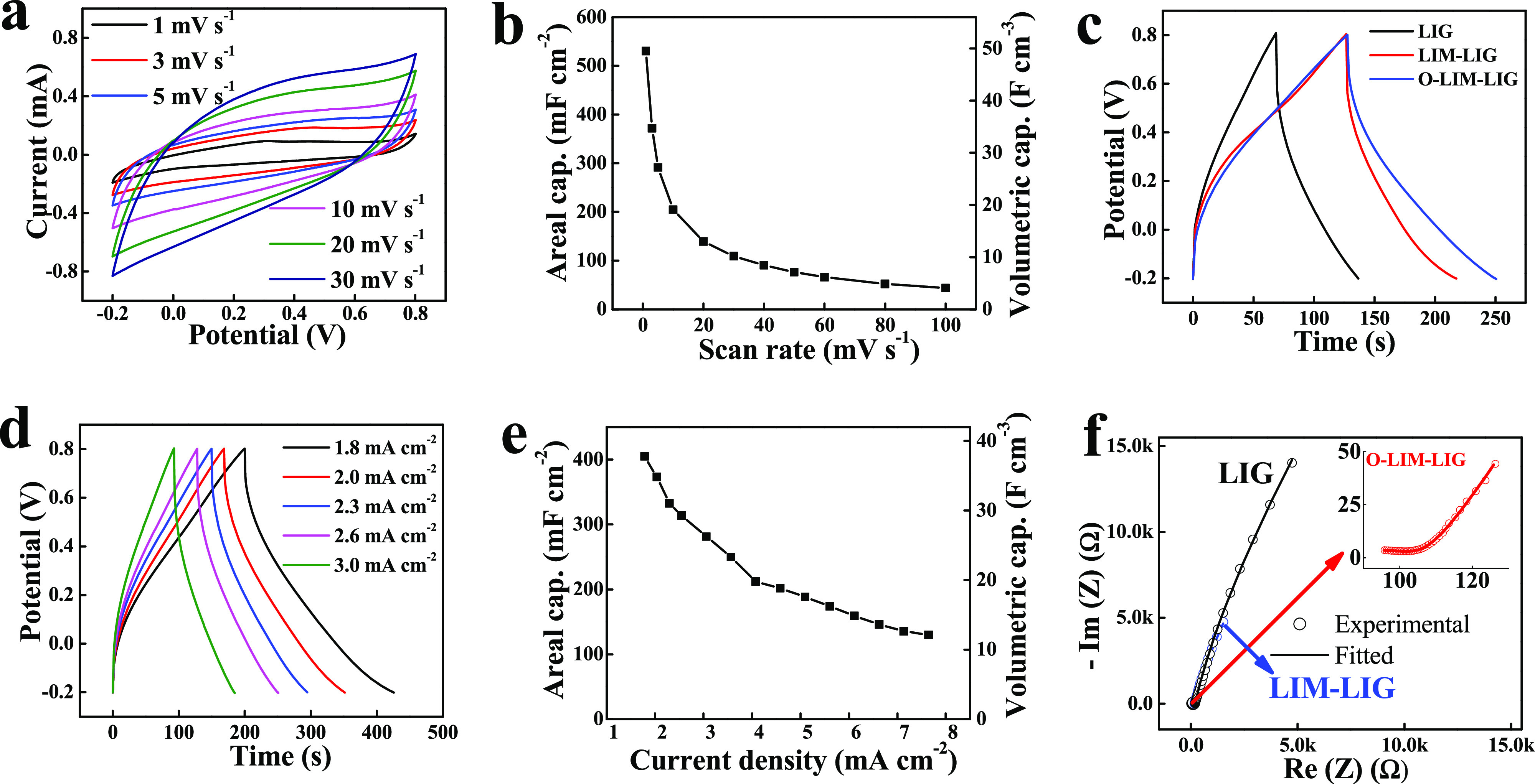
Electrochemical performances of individual electrodes in 1 M H_2_SO_4_ aqueous electrolyte. (a) CV profiles of the O-LIM-LIG at different scan rates and the corresponding (b) areal and volumetric capacitance as a function of scan rate. A quasirectangular CV shape indicates efficient double-layer formation. (c) GCD profile comparison between LIG, LIM-LIG, and O-LIM-LIG at the current density of 2.6 mA cm^–2^. (d) GCD of O-LIM-LIG with varying current density. (e) Evolution of the areal and volumetric specific capacitance of O-LIM-LIG as a function of current density. (f) EIS plots of LIG, LIM-LIG, and O-LIM-LIG with a magnified EIS plot of O-LIM-LIG provided in the inset.

The electrochemical performance was first evaluated in a three-electrode system. Supplementary Figure 10 represents the cyclic voltammetry (CV) profile of LIG, LIM-LIG, and O-LIM-LIG at a scan rate of 20 mV s^–1^. The CV curves exhibit a typical quasirectangular shape without any distinct peaks within the voltage range from −0.2 to 0.8 V, indicating electrical double-layer type charge storage. Notably, the O-LIM-LIG film shows a higher CV integration area as compared to the MXene and LIM-LIG film. The CV curves of O-LIM-LIG SC at various scanning rates (1–100 mV s^–1^) emerged as almost quasirectangular shapes (Figure 5a and Supplementary Figure 11). Note that the O-LIM-LIG micro-SC delivered exceptional areal (*C*_A_ ∼291 mF cm^–2^ at 5 mV s^–1^) and volumetric capacitance (*C*_V_ ∼27 F cm^–3^ at 5 mV s^–1^), both of which are higher than the most reported LIG and MXene-based micro-SCs. The scan rate variation of *C*_A_ and *C*_V_ is shown in Figure 5b with a capacitance retention of around 35% from 20 to 100 mV s^–1^. Figure 5c shows the constant current galvanostatic charge–discharge (GCD) curves of the LIG, LIM-LIG, and O-LIM-LIG at a current density of 2.6 mA cm^–2^. The longest discharge GCD profile reveals the superiority of the O-LIM-LIG over the LIG and LIM-LIG. The triangular GCD shape at different current densities (Figure 5d and Supplementary Figure 12) with a Coulombic efficiency of ∼97% reveals the excellent reversibility of the O-LIM-LIG SC without any noticeable voltage drop at the beginning of the discharge curve. Figure 5e illustrates the *C*_V_ ∼ 35 F cm^–3^ and corresponding *C*_A_ ∼372 mF cm^–2^ at ∼2 mA cm^–2^ outperforming previously reported LIG and MXene-based micro-SCs.

To further investigate the kinetics of ion transport of the LIM-LIG films and pure LIG film, electrochemical impedance spectroscopy (EIS) measurements were performed at open circuit voltage (OCV). As shown in Figure 5f, it is clear to observe that the Nyquist plots of LIG and LIM-LIG deliver similar types of graphs containing a steady proportional increase in the imaginary and real impedance, indicating a sluggish ion diffusion for these electrodes, whereas the O-LIM-LIG showed a steeper slope in the low-frequency region relative to the LIG and LIM-LIG films, which is consistent with the improved ion accessibility and transport due to the additional activation of the O_2_ plasma treatment. Furthermore, the interfacial charge transfer resistance (*R*_ct_) of O-LIM-LIG (∼40 Ω) is evidently less than LIG (∼70 Ω) and LIM-LIG film (∼65 Ω), demonstrating the ionic conductivity of the O-LIM-LIG was improved following O_2_ plasma treatment, which is consistent with the result of CV and GCD testing.

**Figure 6:**
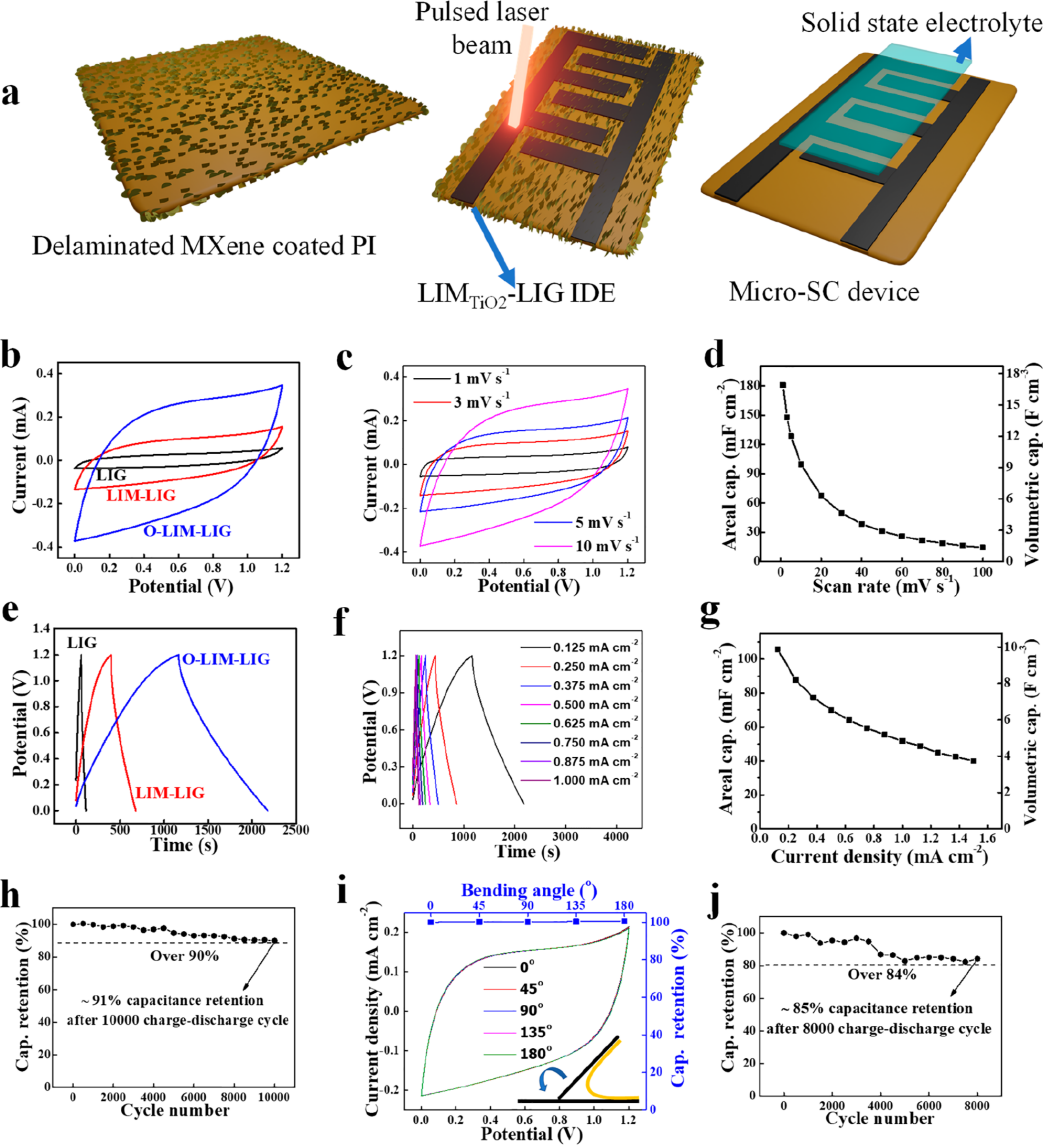
Electrochemical performance of individual micro-SCs in PVA-H_2_SO_4_ gelled electrolyte. (a) Schematic fabrication process of the micro-SC device. Micro-SC device fabricated through laser writing on a delaminated Ti_3_C_2_T_*x*_-coated PI sheet followed by solid-state gel electrolyte coating. (b) Comparative CV curves of LIG, LIM-LIG, and O-LIM-LIG film at a scan rate of 10 mV s^–1^. (c) CV profiles of the corresponding areal and volumetric capacitances as a function of scan rate. (d) Comparative GCD profiles of the LIG, LIM-LIG, and the corresponding O-LIM-LIG at a current density of 0.125 mA cm^–2^. (f) GCD profiles of O-LIM-LIG at different current densities and (g) corresponding areal and volumetric capacitance as a function of current densities. (h) Cyclic stability of O-LIM-LIG. The device retains ∼91% of its initial capacitance after 10 000 charge/discharge cycles. (i) CV profiles of O-LIM-LIG under different bending conditions. The electrochemical performances are unaffected by mechanical deformation. (j) Cyclic stability of O-LIM-LIG under the 180° bending condition. The bent device retains >85% of its initial capacitance after 8000 charge/discharge cycles.

Next, to prepare a solid-state flexible micro-SC device, poly(vinyl alcohol) (PVA)-H_2_SO_4_ polymer gel electrolyte was casted on the interdigitated electrode (IDE) surface without any binder, separator, or any packaging material (see Figure 6a). All devices’ CV curves have a quasirectangular shape (Figure 6b), suggesting strong electrical double-layer (EDL) characteristics. The O-LIM-LIG exhibited the highest *I–V* loop area among the three CV profiles, highlighting the superiority over pure LIG and LIM-LIG in terms of areal and volumetric capacitance value. The CV curves of the O-LIM-LIG micro-SC have an almost quasirectangular shape at a lower scan rate (1 to 10 mV s^–1^), indicating the capacitive behavior of the electrodes (Figure 6c). However, the nature of the CV curve became resistive with increasing scan rate (Supplementary Figure 13), demonstrating the increased effect of the internal resistance of the electrode at a high current density. This is further supported by the large intercept (∼260 Ω) on the axis in the Nyquist plot (Supplementary Figure 15). Nonetheless, its galvanostatic charge–discharge curves (Figure 6e,f) have a triangular shape with a Coulombic efficiency of ∼97%, indicative of the formation of efficient EDL with excellent reversibility and good charge propagation between the interdigitated electrodes. The longest discharge period in the triangular GCD profile at a current density of 0.125 mA cm^–2^ (Figure 6e) further confirmed the superiority of the O-LIM-LIG over the LIG and LIM-LIG. We measured the specific capacitance over a wide range of GCD current density and CV scan rate, respectively. Notably, the O-LIM-LIG micro-SC device delivered exceptional *C*_A_ ∼130 mF cm^–2^ and *C*_V_ ∼12 F cm^–3^ at a scan rate of 5 mV s^–1^ (Figure 6d). Figure 6g illustrates that the volumetric capacitance of the micro-SC (*C*_cell/v_ normalized to the whole device volume) is ∼6.6 F cm^–3^ at 0.5 mA cm^–2^, corresponding to the areal capacitance (*C*_cell/A_) of ∼70 mF cm^–2^ outperforming most reported LIG and MXene-based micro-SCs (Supplementary Table 3). Figure 6h shows that micro-SC retains ∼91% of its initial capacitance even after 10 000 charge–discharge cycles demonstrating its excellent electrochemical stability with long cycle life.

The O-LIM-LIG micro-SC was further subjected to a mechanical bending test to see its adaptability to flexible and wearable electronics. Figure 6i shows that the micro-SC retains ∼100% capacitance compared to its flat state when severely bent. Furthermore, the flexibility endurance test of the micro-SC device was carried out by 8000 GCD cycles by keeping the device bent at 180°. Outstanding cyclic stability was recorded (Figure 6j) with capacitance retention of 85% after 8000 GCD cycles. The high mechanical flexibility of the O-LIM-LIG micro-SC makes it a viable candidate for flexible microelectronics.

Meanwhile, EIS further explains the excellent capacitive performance of the O-LIM-LIG micro-SC (Supplementary Figure 15). EIS measurement shows that in the high-frequency region, O-LIM-LIG (∼260 Ω) has the smallest equivalent series resistance as compared to LIM-LIG (>400 Ω) and LIG (>800 Ω) and displayed a larger slope in the low-frequency region. MXene inclusion minimizes the graphene layer stacking, resulting in a wide surface area with well-defined mesoporosity for effective electrolyte penetration and ion adsorption. Meanwhile, the presence of oxygen functional groups improves the polar interaction with the electrolyte solution.^7^

**Figure 7:**
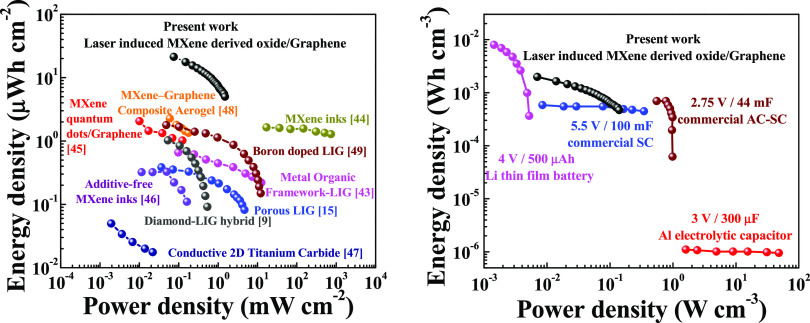
Ragone plot of the O-LIM-LIG micro-SC where energy and power densities are compared with the state-of-the-art LIG and MXene-based energy storage systems and commercially available supercapacitors. Volumetric energy and power densities of O-LIM-LIG micro-SC compared with commercial supercapacitors and a 4 V/500 μAh Li film battery.^7,21,54^ Data for the Li battery are reproduced from ref (3). Data for the 2.75 V/44 mV activated carbon supercapacitor, 5.5 V/100 mF commercial supercapacitor, and 3 V/300 μF Al electrolytic capacitor are reproduced from refs (21) and (49).

The Ragone plot (Figure 7) further showcases the potential of the O-LIM-LIG interdigitated electrodes for high energy/power density micro-SCs. Our micro-SCs have an areal energy density of ∼21.2 μWh cm^–2^ and a power density of 0.075 mW cm^–2^ at a current density of 0.125 mA cm^–2^. By increasing the current density to 10-fold (1.25 mA cm^–2^), almost 45% of the energy density has been retained (∼9 μWh cm^–2^), while the power density increases to 0.75 mW cm^–2^. The energy and power density values are higher than the recently reported SCs based on MXene and laser-induced graphene.^9,15,47−53^ To our knowledge, the areal energy density in this work is one of the highest among all MXene and LIG-based solid-state micro-SCs reported until date. Furthermore, we compare our micro-SCs’ volumetric energy/power performance to that of commercially available energy storage devices. Importantly our micro-SCs outperformed these commercial energy storage devices in terms of the volumetric energy density.

### Flexible Energy Storage Devices for Portable Electronics and Real-Time Health Monitoring System

**Figure 8:**
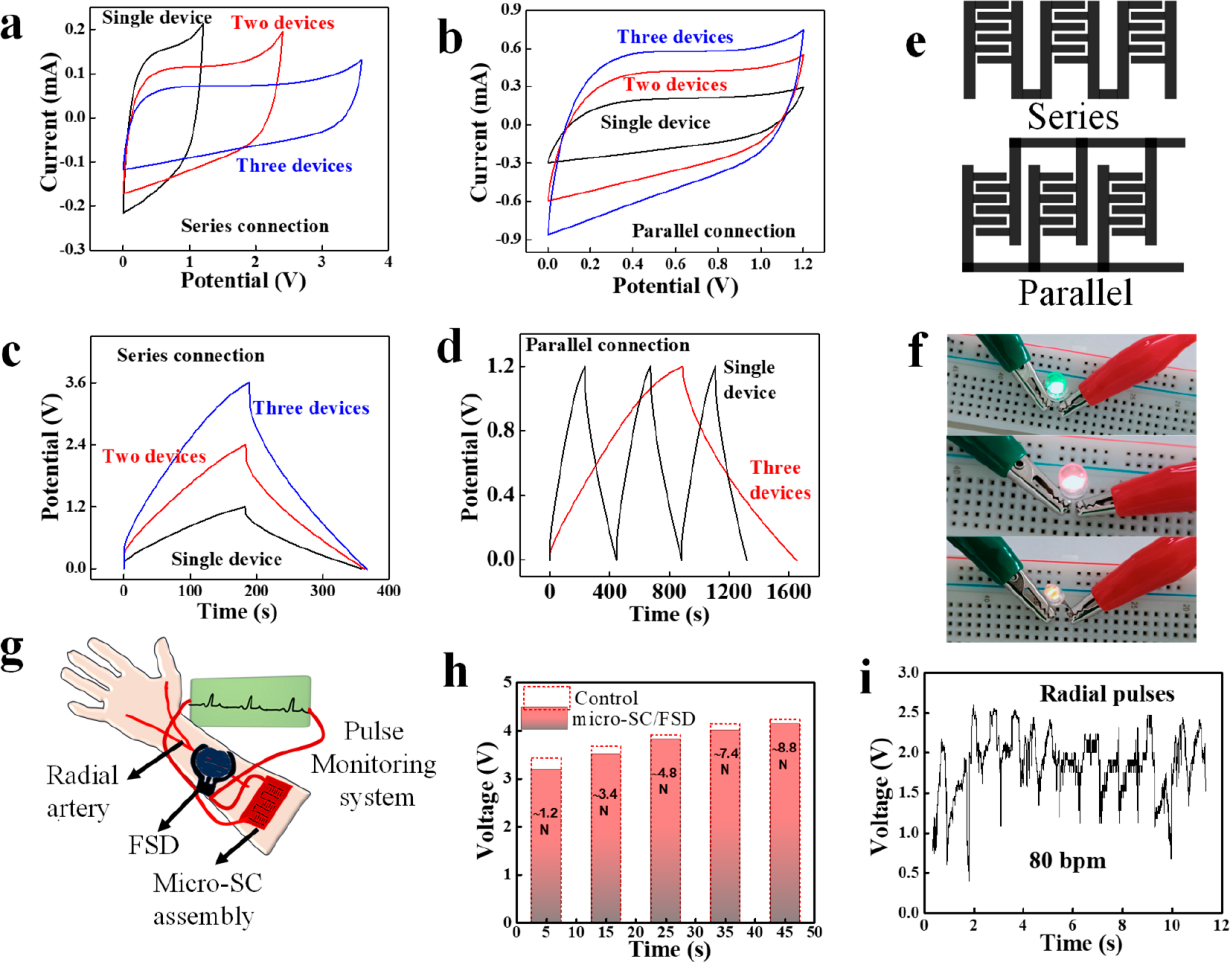
Application of micro-SC devices connected in series and parallel conditions. CV curves at 5 mV s^–1^ and GCD profiles at 0.5 mA cm^–2^ of O-LIM-LIG micro-SC devices connected in (a,c) series and (b,d) parallel. (e) Schematic of the series and parallel combinations of three micro-SC electrodes. (f) Photographs of commercial red, yellow, and green LEDs powered by micro-SC devices connected in series. (g) Schematic of powering the FSD to monitor the human body radial artery pulses using wearable micro-SC devices. (h) Force-dependent voltage response of FSD powered by serially connected micro-SC (micro-SC/FSD) and an external 5 V power supply (control). (i) Recorded live radial pulses of a human body around ∼80 BPM.

In order to meet the desired power/energy standards, portable electronics often required cells assembled either in series, in parallel, or a combination of the two. Thus, it would be interesting to develop a device that has control over the operating voltage and current by using series and parallel combinations of EC cells with minimal energy losses. To meet the specific energy/power output, three micro-SCs were connected in both series (Figure 8a–d) and parallel. In comparison to a single micro-SC with an operating potential of 1.2 V, three micro-SCs linked in series generated 3.6 V CV (Figure 8a) and a charge–discharge window with a similar discharge time (Figure 8c). When operated at a parallel configuration, the output current of three parallel linked micro-SCs rose by a factor of 3 (Figure 8b), and their discharge duration was three times that of a single micro-SC (Figure 8d). To demonstrate its applicability in the portable electronics sector, we constructed three micro-SC units in series to power commercial electronics including LEDs, a digital watch, a force-sensitive detector, etc. Notably, in series, our devices could readily power LEDs for more than 2 min (Supplementary Movies S1, S2, and S3). The device can also support the regular and persistent operation of digital watches and digital temperature sensor displays (Supplementary Movies S4 and S5). The micro-SCs’ capacity to power commercial electronic devices indicates their immense potential for usage in powering electronics.

The ideal way to develop a future smart biomonitoring system is to integrate it into flexible and miniaturized portable energy storage devices. The FSD is a polymeric sheet that records the current/voltage response as a function of applied force. As seen in Figure 8h, the voltage response steadily increases as the force applied to the FSD increases when the FSD is connected to an external 5 V power source (control). When the FSD is powered by serially connected micro-SCs, the voltage response follows a similar pattern. These results revealed the feasibility of employing the O-LIM-LIG micro-SCs as an alternative for commercial energy storage devices for a real-time biomonitoring system. As a proof of concept, the FSD which was powered by the flexible serially linked O-LIM-LIG micro-SCs is attached to the wrist of a human body to monitor artery pulses (Figure 8g). As illustrated in Figure 8i, the FSD attached to the wrist records the real-time radial pulses of a human body with a heart rate of ∼80 beats per minute (BPM). To validate our findings, we compared our biomonitoring system with a commercial fitness wristband.

### Mechanism of Improved Microsupercapacitor Device Performance

Using a picosecond pulsed laser, a single-step scalable technique is described here to produce in situ MXene (Ti_3_C_2_T_*x*_)-derived Ti-rich oxide nanoparticles attached uniformly to LIG. The carbonized steam from the PI surface was converted to −C=OH^+^ and C–OH_2_^+^ in the presence of abundant oxygen-containing groups on the surface of Ti_3_C_2_T_*x*_ MXene. As the reaction continues to degrade MXene, Ti–O^–^ was linked with the C by making a bond with −C=OH^+^ and C–OH_2_^+^.^55^ As an outcome, the Ti–O–C covalent bonding was formed, which is critical for hybrid architecture assembly. This in situ bonding reinforces the linking between the LIM and LIG interfaces and enhances charge transfer.

These techniques enable the fabrication of miniaturized microscale devices and potentially roll-to-roll manufacturing without the use of any organic binders, conductive additives, or separators, which are frequently required for commercial SCs, resulting in improved device performance due to the ease with which ions can access the electrode material. The laser IR energy generates a local high temperature (∼2000 K) on a Ti_3_C_2_T_*x*_-coated PI sheet, causing a carbonized steam to form in a submillisecond time scale. The stacked Ti_3_C_2_ sheets are degraded and fragmented by the high-temperature thermal oxidation process, resulting in spherical Ti-rich oxide nanoparticles. This process was also corroborated by MD simulations, which displayed the formation of spherical nanoparticles covalently bound (Ti–O–C) to graphene.

The enhanced electrochemical capabilities of the O-LIM-LIG are evident in both aqueous and polymer gel electrolytes and can be attributed to a set of factors. These include the three-dimensional porous structure inherent in LIG, the electrochemically stable spherical particles enriched with titanium, and the introduction of surface defects achieved through a dual combination of laser irradiation and O_2_ plasma treatment. The integration of MXene onto the LIG framework serves a dual purpose: it ensures the structural integrity of the LIM-LIG hybrid structure by preventing self-aggregation and facilitates efficient charge transfer between the LIM and LIG through the formation of Ti–O–C covalent bonds.

Furthermore, the introduction of oxygen-containing groups via the O_2_ plasma treatment enhances the hydrophilicity of the O-LIM-LIG, fostering improved interaction with the electrolyte. This improved wettability not only enhances the contact between the electrode surface and the electrolyte but also facilitates effective capillary action within the micropores, consequently amplifying the accessible surface area for ions.^9,56,57^ Additionally, an upsurge in defect levels after O_2_ plasma treatment (discerned from Raman analysis) plays a role in strengthening the charge storage capacity, consistent with prior research.^9,15,57^

The combination of microscale design with uniform LIM decoration over graphene sheets with a surface fully accessible for electrolyte ions due to post-O_2_ plasma treatment is responsible for the ultrahigh-energy performance of the O-LIM-LIG micro-SC.

## Conclusions

In summary, a single-step roll-to-roll manufacturing technique is described here to produce laser-induced MXene-derived Ti/O-rich nanoparticles covalently bound to graphene. The formation of stable nanoarchitecture at an elevated lasing temperature (∼2000 K) is supported by the MD simulations studies which confirm the formation of a Ti–O–C covalent bond at the MXene-graphene interface. This stable capacitor electrode has a specific areal capacity of ∼373 mF cm^–2^ @ 2 mA cm^–2^ in 1 M H_2_SO_4_ (three-electrode cell) and ∼88 mF cm^–2^ @ 0.25 mA cm^–2^ in PVA/H_2_SO_4_ (two-electrode cell) with an ultrahigh energy density of 21.16 × 10^–3^ mWh cm^–2^, which is about 10 times larger than commercially available SCs and even comparable to the 4 V/500 μAh thin-film lithium battery. Furthermore, the O-LIM-LIG micro-SC exhibits excellent cyclic stability (91% retention after 10 000 cycles), which is an important aspect when compared to microbatteries whose lifetime is a major issue when integrated into biomedical implants, RFID tags, or microsensors where replacement or maintenance is not possible. Our micro-SCs can be directly integrated into any such biomedical device and can make a more efficient self-powered real-time health monitoring system. As a proof of concept, we integrate our micro-SCs with an FSD (used to monitor the real-time biosignal from the human body) to record the radial artery pulses of a human body (∼80 BPM) and verify the results with a commercially available fitness wristband. Our micro-SCs can also be connected in series or parallel configurations to meet the energy/power requirements of portable electronic devices, as exemplified in our study by powering various LEDs and digital watches. For miniaturized portable electronics, our device could bridge the energy density gap between microbatteries and micro-SCs. In the future, to extract energy more efficiently from solar, thermal, and mechanical sources, our O-LIM-LIG micro-SC can be directly integrated on-chip.^58^

## Materials and Methods

### Materials

Exfoliated Ti_3_C_2_T_*x*_ MXene powders were purchased from Laizhou Kai Kai Ceramic Materials Co. Ltd. (Hong Kong S.A.R.). Polyimide sheets (PI, 0.005″) were used as received from Fiedler Scientific Instruments, Czech Republic. Poly(vinyl alcohol), sulfuric acid (H_2_SO_4_, 96%), and dimethyl sulfoxide (DMSO) were purchased from Sigma-Aldrich (Merck, Germany). Glassy carbon, platinum, and Ag/AgCl electrodes were purchased from CH Instruments, Texas, USA. Force sensing devices were purchased from Conrad Electronic, Czech Republic. All materials were used without any further purification/modification.

### Delamination of Ti_3_C_2_T_*x*_ MXene

Delamination was done using dimethyl sulfoxide (DMSO) as intercalant resulting in an increased interlayer spacing of Ti_3_C_2_T_*x*_ MXenes.^59^ First Ti_3_C_2_T_*x*_ powders were dispersed in DMSO followed by 24 h stirring at room temperature. The colloidal suspension was then centrifuged (4000 rpm, 5 min) to separate the intercalated Ti_3_C_2_T_*x*_ powder. After collecting the supernatant, DI water was added followed by bath sonication for 6 h. Again, centrifugation was carried out, and the sediments were collected using vacuum-based filtration and vacuum drying at ∼70 °C overnight.

### Fabrication of the MXene-LIG Hybrid

An MXene-LIG hybrid electrode was prepared by a diode-pumped solid-state Nd:YAG laser (Laser dicer Oxford Lasers A-Series) working at 532 nm wavelength. First, the delaminated Ti_3_C_2_ MXene was spin-coated onto the polyimide sheet (500 rpm, 60 s). The defocused laser writing method on a Ti_3_C_2_ MXene-coated PI sheet results in an MXene-LIG hybrid electrode. Detailed laser (diode-pumped solid-state Nd:YAG, 532 nm) parameters are as follows: Laser power, 3 W, scanning speed 50 mm/s, pulse frequency 7 kHz, resolution 0.5 μm, central wavelength, 532 nm.

### Fabrication of Flexible MSC

The solid-state supercapacitors were patterned into 10 interdigitated electrodes (IDEs) with the dimensions of 1 mm (*h*) × 5 mm (*w*) and a 300 μm gap between two IDEs. Following that, a polymeric hydrogel (PVA/H_2_SO_4_) electrolyte was prepared by mixing 1 g of PVA with 10 mL of DI water and stirring continuously for 3 h at 80 °C before adding 1 mL of sulfuric acid dropwise to create a transparent gel-like electrolyte. Finally, the PVA/H_2_SO_4_ hydrogel was uniformly applied to the IDE before being used as a microsupercapacitor device.

### Materials Characterization

The surface morphologies and cross-sectional images were captured using FEI VERIOS 460L. The corresponding elemental mapping was taken with an EDX detector (EDAX SDD Octane Super) attached along with the FEI VERIOS 460L. Surface chemical compositions were studied by XPS using a Kratos Analytical Axis Supra instrument with a monochromatic Al Kα (1486.7 eV) excitation source. All XPS spectra were calibrated to the adventitious C 1s peak at 284.8 eV and fitted with CasaXPS software. The Raman spectra of the material were recorded within a range of 200–3000 cm^–1^ by using a Raman spectrometer (Witec Alpha 300R) with a 532 nm laser. The sheet resistance of the samples was measured at room temperature using a four-probe setup. The contact angle was measured using See System E Advex Instruments. The CLSM (Olympus Lext OLS4100) was used to estimate the surface roughness and height profiles of the films where a laser light source of a wavelength of 405 nm was used during the CLSM experiment.

### Electrochemical Measurements

The electrochemical measurements were investigated by cyclic voltammetry (CV), electrochemical impedance spectroscopy (EIS, frequency range of 0.01 Hz–100 kHz with a 5 mV ac amplitude), and galvanostatic charge–discharge (GCD) measurement using a potentiostat-galvanostat (Metrohm Autolab, PGSTAT 204, Netherlands) equipped with the Nova 2.1.4 software. Three-electrode tests were carried out in 1 M H_2_SO_4_ using Ag/AgCl in 1 M KCl as the reference electrode, Pt as the counter electrode, and O-LIM-LIG as the working electrode.

Specific areal capacitance (*C*_A_) of the MSC devices was calculated from CV and GCD measurements according to eqs 1 and 2,
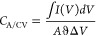
1where *∫I(V)dV* is the integrated area from the discharge part of CV curves, *A* is the geometrical surface area of the IDE, ϑ is the scan rate, and Δ*V* is the voltage window.
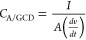
2where *I* is the discharge current, *A* is the geometrical surface area, and  is the slope of the discharge curve.

Specific volumetric capacitance (*C*_V_) is calculated by the following equation,
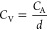
3where *d* is the thickness of the O-LIM-LIG hybrid.

The areal energy density (*E*_A_) and volumetric energy density (*E*_V_) of the MSCs were calculated by using eqs 4 and 5, respectively:
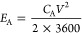
4
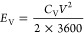
5where *V* is the applied potential.

The areal power density (*P*_A_) and volumetric power density (*P*_V_) of the MSCs were calculated by using eqs 6 and 7, respectively:
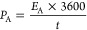
6
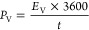
7where *t* is the discharge time.

### Simulation Setup

ReaxFF reactive force field as implemented in the Large Scale Atomistic/Molecular Massively Parallel Simulator (LAMMPS; version from 10 Aug 2015) was employed for simulation of interaction of MXene with graphene.^45,60^ Details of the considered method could be found elsewhere.^61^ For the simulation, we selected force field parameters that matched those for O-terminated MXene, which have previously been successfully applied in water/surface studies of heterostructures including MXenes.^46^ The used models (composed of C, Ti, and O atoms) were constructed by taking into account that the graphene surface was already formed because the graphitization process is a long-lasting process that we are not able to simulate on achievable time scales. We focused on the formation and behavior of MXene nanoparticles on graphene surfaces at high temperatures (2000 and 2500 K) generated by the laser pulse. Ti_3_C_2_O MXene 18.2 × 15.8 Å long was positioned 8 Å above a periodic graphene surface with dimensions of 38.8 × 37.8 Å. Various starting models were constructed including (i) the MXene divided into four smaller pieces, (ii) graphene with variable flexibility (fixed/artificially wrinkled graphene), and (iii) additional oxygen atoms added on the graphene surface (modeling airborne oxygen radicals formed at the high temperature). In total, eight independent simulations were executed with a total length of 560 ps for each configuration. The system was initially minimized for 1000 steps and then subjected to a 50 ps NVT-MD heat-up simulation from 300 to 2000/2500 K. Following this, the system was allowed to evolve for 300 ps at 2000/2500 K and then cooled to 300 K over an additional 200 ps. All molecular dynamics simulations were performed using a time step of 0.1 fs and velocity-rescale thermostat (with a damping constant of 0.1 ps).^62^

## Supporting Information Available

Additional characterization data like SEM, XPS, water contact angle, sheet resistance (Figures S1 to S8). Additional theoretical simulation result (Figure S9). Additional electrochemical results and EIS (Figures S10 to S15). Raman and XPS fitting results (Tables S1 and S2). MSC device performance with the current state of art (Table S3) (PDF)
Movie S1: Device powering LED for more than 2 min (MP4)
Movie S2: Device powering LED for more than 2 min (MP4)
Movie S3: Device powering LED for more than 2 min (MP4)
Movie S4: Device supporting the regular and persistent operation of digital watches and digital temperature sensor displays (MP4)
Movie S5: Device supporting the regular and persistent operation of digital watches and digital temperature sensor displays (MP4)
