# Supplementary material for: Laser-Induced MXene-Functionalized Graphene Nanoarchitectonics-Based Microsupercapacitor for Health Monitoring Application
Source: ACS Nano. 2023 Oct 4;17(20):20537–50. doi: 10.1021/acsnano.3c07319 (PMC10604107; doi:10.1021/acsnano.3c07319)
Supplement: Supplementary file 1 — nn3c07319_si_001.pdf [file nn3c07319_si_001.pdf]

Supplementary Information

Laser Induced MXene Functionalized Graphene Nanoarchitectonics based Microsupercapacitor for Health Monitoring Application

Sujit Deshmukh[1], Kalyan Ghosh[1], Martin Pykal,[2] Michal Otyepka[2,3] & Martin Pumera [1, 4, 5, 6]

[1] Future Energy and Innovation Laboratory, Central European Institute of Technology, Brno University of Technology, Purkyňova 123, 61200 Brno, Czech Republic.

[2] Regional Centre of Advanced Technologies and Materials, Czech Advanced Technology and Research Institute (CATRIN), Palacký University in Olomouc, Šlechtitelů 27, 783 71, Olomouc, Czech Republic.

[3] IT4Innovations, VŠB-Technical University Ostrava, 17. listopadu 2172/15, 708 00 Ostrava-Poruba, Czech Republic.

[4] Faculty of Electrical Engineering and Computer Science, VSB - Technical University of Ostrava, 17. listopadu 2172/15, 70800 Ostrava, Czech Republic

[5] Department of Chemical and Biomolecular Engineering, Yonsei University, 50 Yonsei-ro, Seodaemun-gu, Seoul 03722, Korea

[6] Department of Medical Research, China Medical University Hospital, China Medical University, No. 91 Hsueh-Shih Road, Taichung 40402, Taiwan

🖂email: martin.pumera@ceitec.vutbr.cz

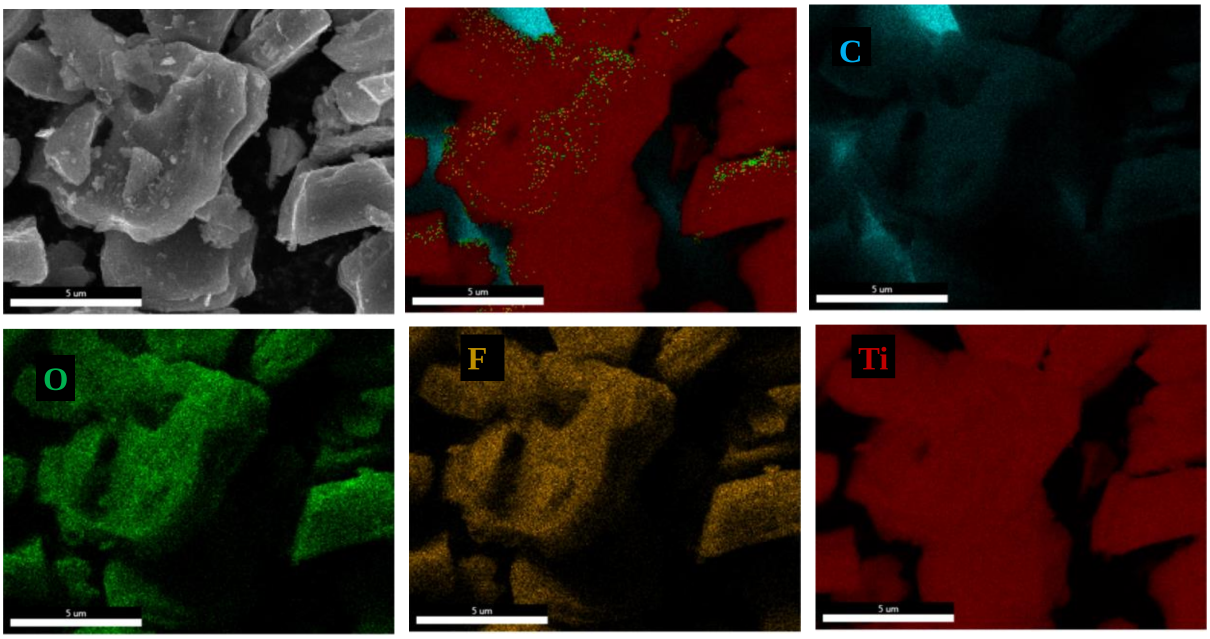

Supplementary Figure 1. Elemental mapping images of $Ti_3C_2T_x$ MXene revealing the presence of Ti, O, F, and C. Scale bar 5 µm.

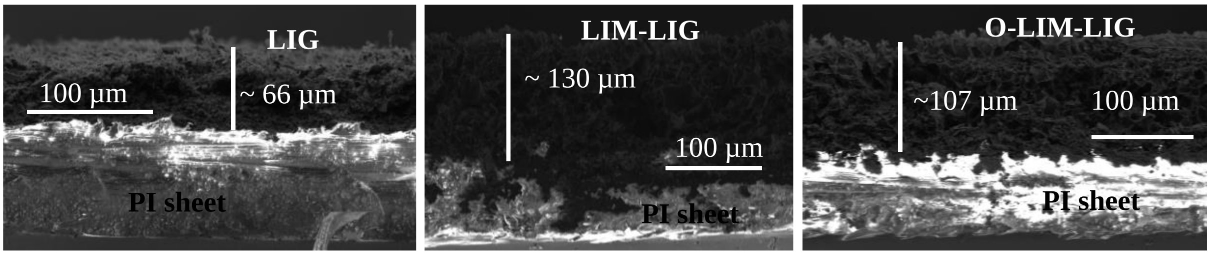

Supplementary Figure 2. Cross-section SEM images of LIG, LIM-LIG, and O-LIM-LIG film. In comparison to LIG, rapid thermal oxidation of $Ti_3C_2T_x$ MXene increases the porosity of the LIM-LIG hybrid.

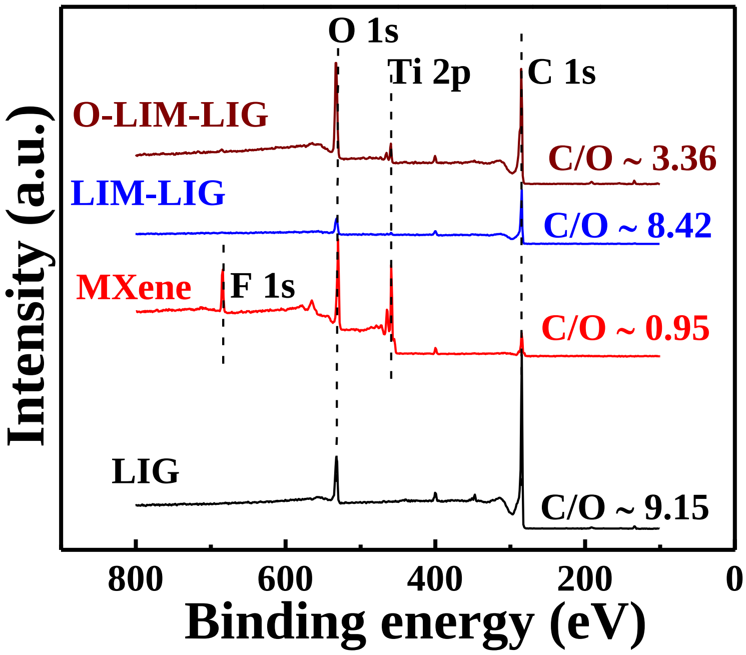

Supplementary Figure 3. XPS survey spectra of LIG, delaminated MXene, LIM-LIG, and O-LIM-LIG film surface. The C/O ratio is maximum for LIG and minimum for O-LIM-LIG.

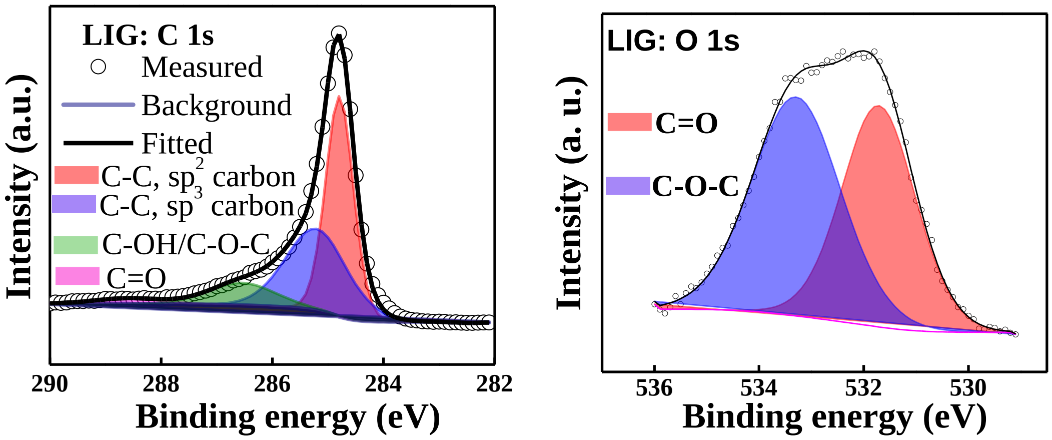

Supplementary Figure 4. C 1s, and O 1s high-resolution XPS spectra of LIG. The C 1s XPS spectrum of LIG exhibited the peaks (C-C sp^2 and sp^3 carbon, C=OH, and C=O), consistent with the previous report.[1]

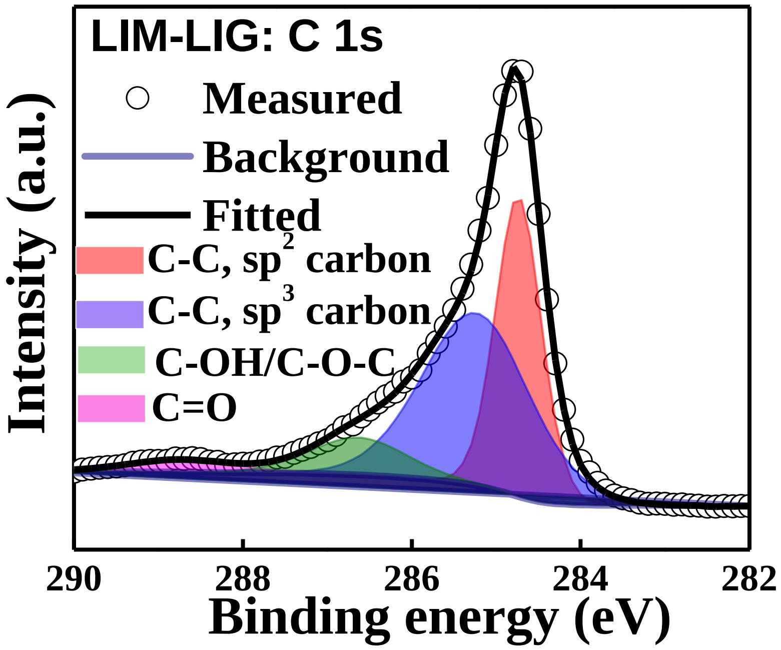

Supplementary Figure 5. C 1s high-resolution XPS spectra of LIM-LIG.

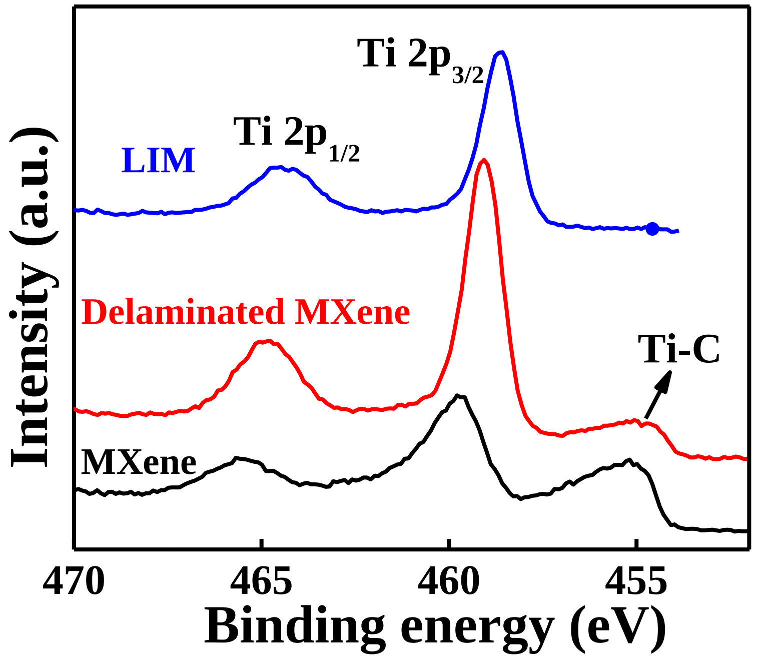

Supplementary Figure 6. Ti 2p high-resolution XPS spectra of MXene, delaminated MXene and LIM.

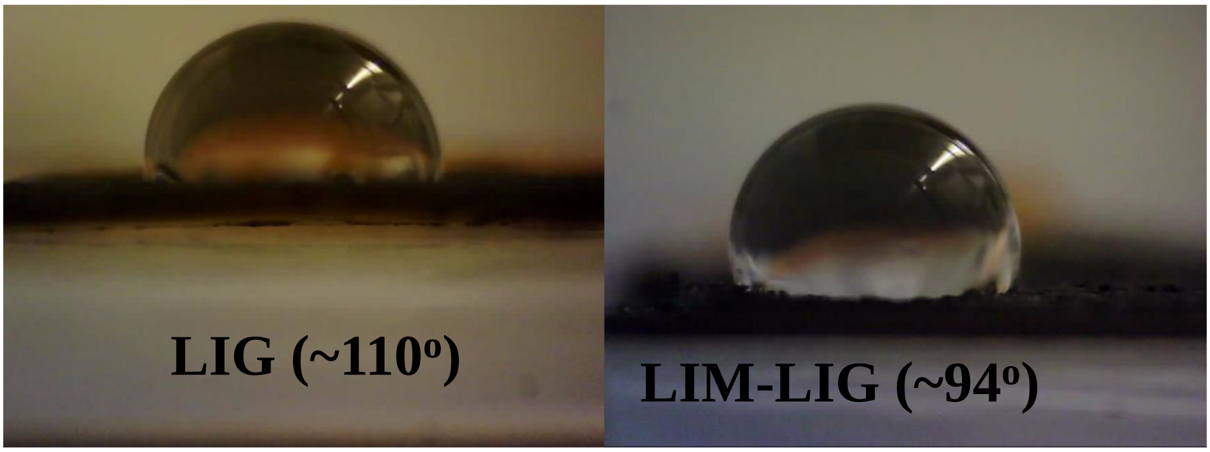

Supplementary Figure 7. Snapshots of the water droplets on the LIG and LIM-LIG surface revealing both surfaces are hydrophobic (water contact angle > 90°).

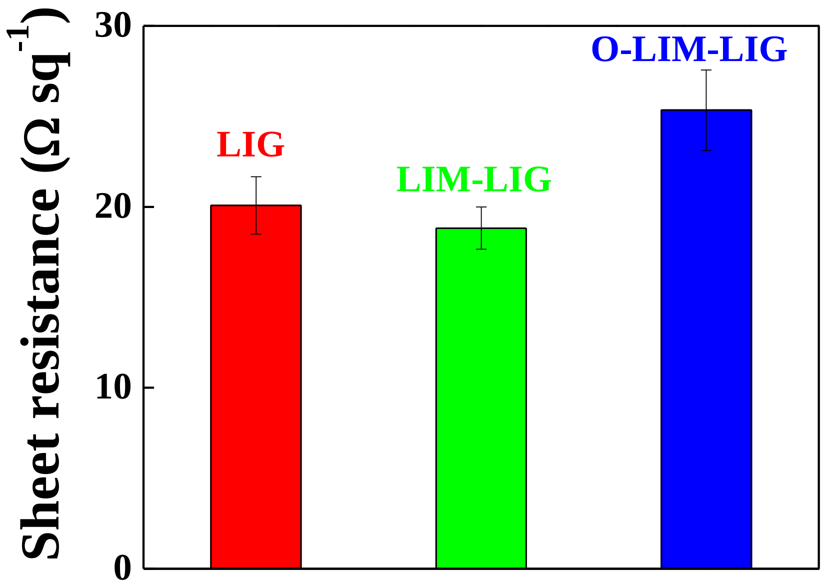

Supplementary Figure 8. Sheet resistance comparison between LIG, LIM-LIG, and O-LIM-LIG film.

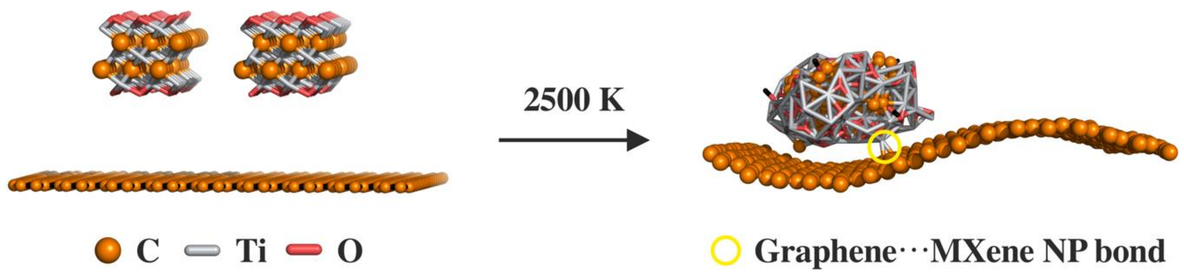

Supplementary Figure 9. Initial and final snapshots taken from ReaxFF simulation at elevated temperature 2500 K showing the formation of covalently bound MXene-based nanoparticles on graphene surface. The covalent intermolecular bonds are highlighted by yellow circle.

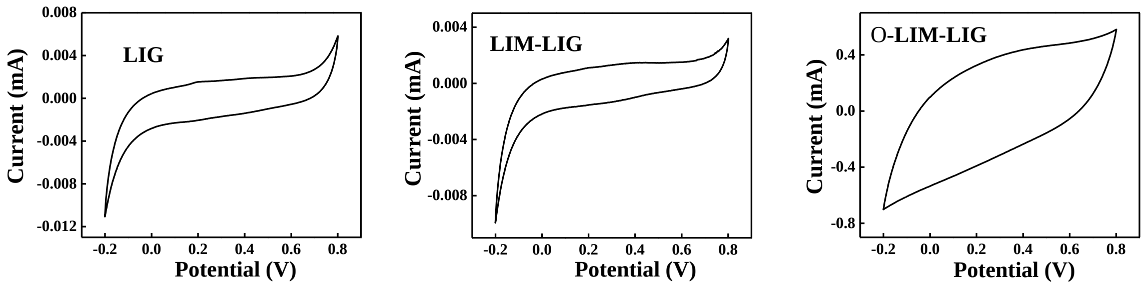

Supplementary Figure 10. CV profiles of LIG, LIM-LIG, and O-LIM-LIG @ 20 mV s^{-1}. Electrolyte used 1 M H_2SO_4.

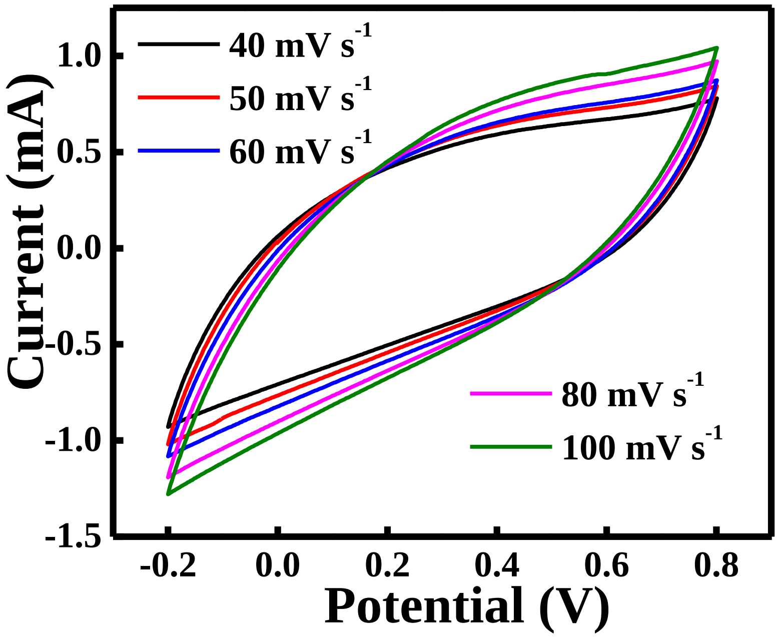

Supplementary Figure 11. CV profiles of O-LIM-LIG at different scan rates. Electrolyte used 1 M H_2SO_4.

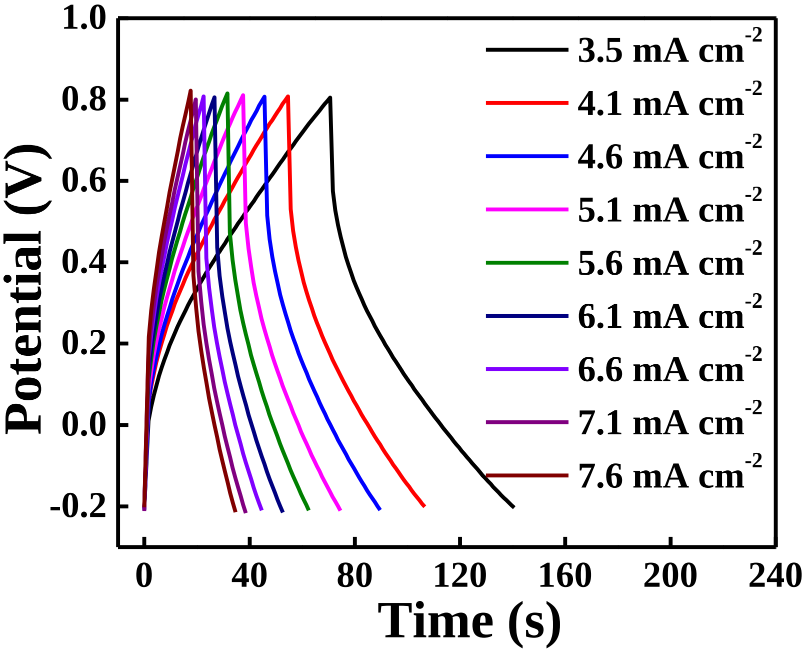

Supplementary Figure 12. GCD profiles of O-LIM-LIG at different current densities. Electrolyte used 1 M H_2SO_4.

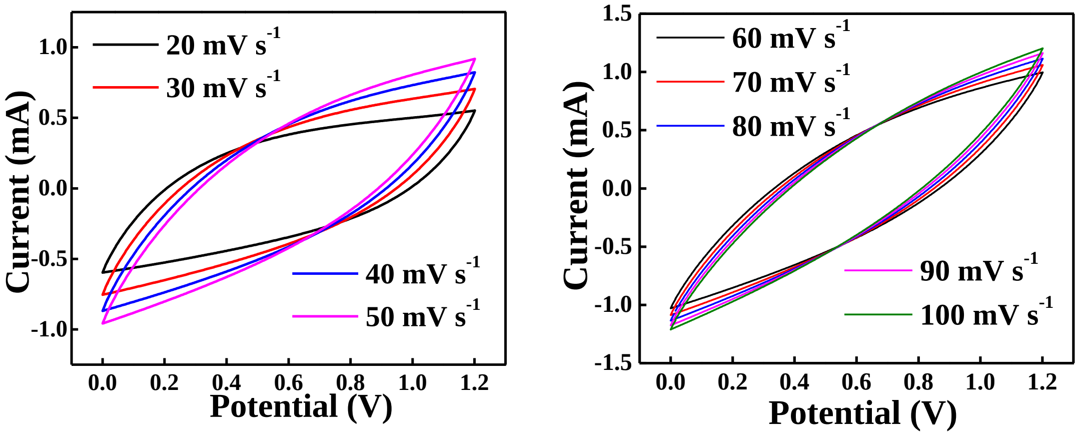

Supplementary Figure 13. CV profiles of O-LIM-LIG micro-SC at different scan rates. Electrolyte used; PVA/H_2SO_4 gel.

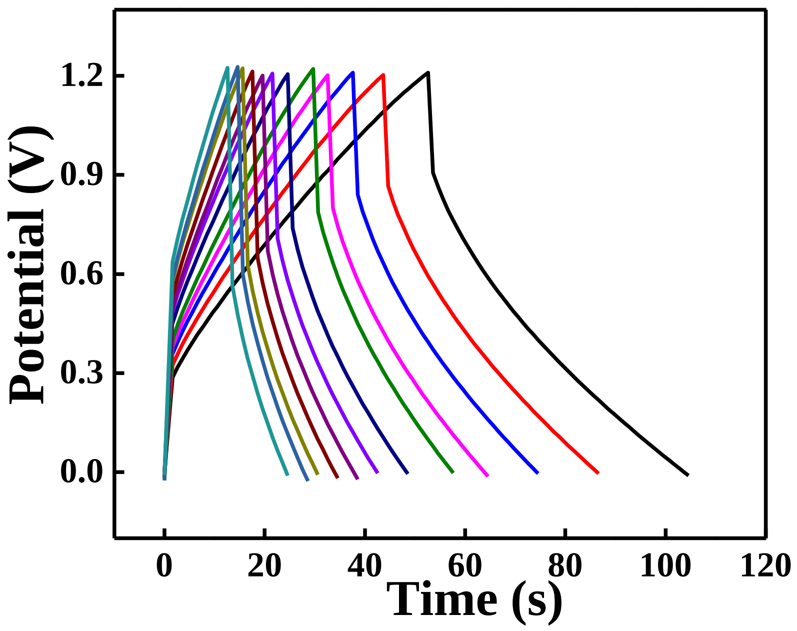

Supplementary Figure 14. GCD profiles of O-LIM-LIG micro-SC at different current densities (1.125 to 2.5 mA cm^{-2}). Electrolyte used; PVA/H_2SO_4 gel.

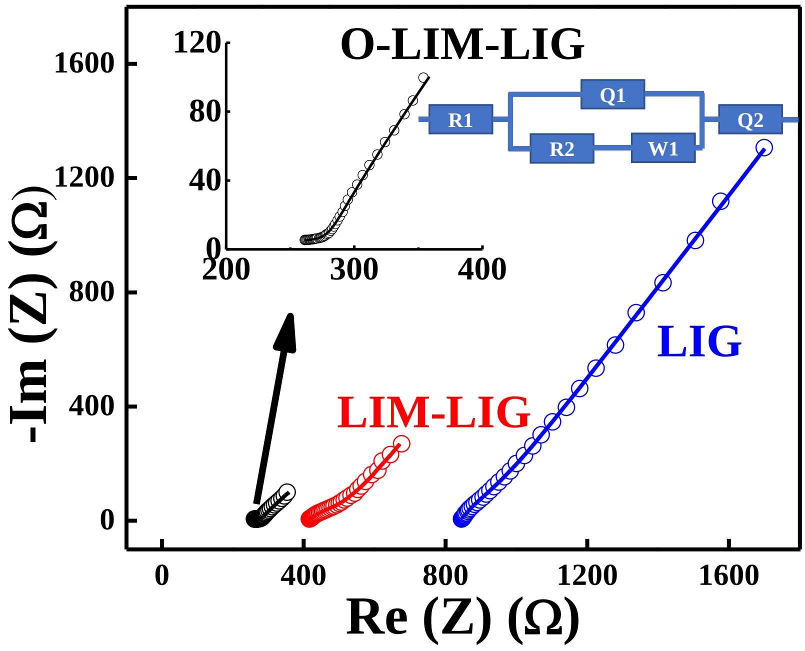

Supplementary Figure 15. Nyquist plots of LIG, LIM-LIG, and O-LIM-LIG micro-SC with the corresponding equivalent circuit model. The magnified view of O-LIM-LIG is shown in the inset.

Supplementary Table 1. Raman fitting results.

Sample	D band position (cm^{-1})	FWHM D band (cm^{-1})	G band position (cm^{-1})	FWHM G band (cm^{-1})	2D band position (cm^{-1})	FWHM 2D band (cm^{-1})	I_D/I_G	$I_D/I_{D'}$	I_{2D}/I_G
LIG	1339.91	50.11	1577.02	50.01	2673.80	86.77	0.78	-	0.80
LIM-LIG	1339.24	40.42	1568.92	26.27	2676.09	52.58	0.87	5.56	1.06
O-LIM-LIG	1338.43	50.16	1568.97	33.66	2675.80	62.91	1.19	3.84	0.75

Supplementary Table 2. XPS fitting results.

Sample	Region	Binding energy(eV)	Peak assignment	Ref
LIG	C 1s	284.79	C–C (sp^2)	[1]
		285.21	C–C(sp^3)	
		286.54	C–OH/C–O–C	
		288.59	C=O	
	O 1s	531.71	C=O	
		533.29	C–O–C	
$Ti_3C_2T_x$ MXene	Ti $2p_{3/2}$ (Ti $2p_{1/2}$)	454.76 (460.46)	Ti–C (MXene)	[2] [3]
		456.76 (461.86)	Ti^{3+}	
		459.06 (464.76)	TiO_2	
	C 1s	281.2	C–Ti–Tx	
		284.8	C–C	
		286.47	CH_x/C–O	
		288.97	O–C=O	
	O 1s	530.6	TiO_2	
		531.9	C–Ti–O_x	
		533.6	H_2O_{abs}	
O-LIM-LIG	Ti $2p_{3/2}$	459.5	Ti $2p_{3/2}$	[2] [3]
	Ti $2p_{3/2}$	465.3	Ti $2p_{1/2}$	
	C 1s	284.74	C–C (sp^2)	
		285.04	C–C (sp^3)	
		286.57	C–OH	
		287.45	C=O	
		289.25	O-C=O	
	O 1s	529.92	TiO_2	
		531.14	C–Ti–O_x	

Supplementary Table 3. Comparison of our MSC device performance with the current state of art.

Electrode material and electrolyte used	$C_{A/CV}$ (mF cm^{-2})	$C_{A/GCD}$ (mF cm^{-2})	Ref
Laser-sculptured ultrathin transition metal carbide<br>PVA/LiTFSI	20@1 mV s^{-1}<br>2@100 mV s^{-1}		[4]
MXene/rGO<br>PVA/H_3PO_4	372.2@10 mV s^{-1}		[5]
MXene−Graphene Composite Aerogel<br>PVA/H_2SO_4	34.6 @1 mV s^{-1}		[6]
MXene Quantum Dots/Graphene<br>PVA/H_2SO_4	64.2@1 mV s^{-1}	65.2@0.6 mA cm^{-2}	[7]
MXene Sediment Inks<br>PVA/H_2SO_4	~120@10 mV s^{-1}	158@0.08 mA cm^{-2}	[8]
BDNW decorated oxidized LIG<br>PVA/H_2S_{O4}	25.2@1 mV s^{-1}	9.4@0.1 mA cm^{-2}	[1]
Ruthenium oxide-based LIG<br>PVA/H_2SO_4	16@20 mV s^{-1}	9.54@0.1 mA cm^{-2}	[9]
LIG prepared from PI films<br>1M H_2SO_4	4.1@1 mV s^{-1}	3.9@0.2 mA cm^{-2}	[10]
3D Printing of Freestanding MXene<br>PVA/H_2SO_4		2.1 F cm^{-2} @1.7 mA cm^{-2}	[11]
MOF-LIG (MOF-199@ZIF-67)<br>1M H_2SO_4	8.1@1 mV s^{-1}<br>6.2@10 mV s^{-1}	5.0@0.2 mA cm^{-2}	[12]
Laser-induced MXene decorated LIG<br>PVA/H_2SO_4	128.6@5 mV s^{-1}	87.7@0.25 mA cm^{-2}	Present work

References

(1) Deshmukh, S.; Jakobczyk, P.; Ficek, M.; Ryl, J.; Geng, D.; Bogdanowicz, R. Tuning the Laser-Induced Processing of 3D Porous Graphenic Nanostructures by Boron-Doped Diamond Particles for Flexible Microsupercapacitors. *Adv. Funct. Mater.* **2022**, *n/a* (n/a), 2206097. https://doi.org/https://doi.org/10.1002/adfm.202206097.

(2) Urso, M.; Ussia, M.; Novotný, F.; Pumera, M. Trapping and Detecting Nanoplastics by MXene-Derived Oxide Microrobots. *Nat. Commun.* **2022**, *13* (1), 3573. https://doi.org/10.1038/s41467-022-31161-2.

(3) Low, J.; Zhang, L.; Tong, T.; Shen, B.; Yu, J. TiO_2/MXene Ti_3C_2 Composite with Excellent Photocatalytic CO2 Reduction Activity. *J. Catal.* **2018**, *361*, 255–266.

https://doi.org/https://doi.org/10.1016/j.jcat.2018.03.009.

(4) Zang, X.; Jian, C.; Zhu, T.; Fan, Z.; Wang, W.; Wei, M.; Li, B.; Follmar Diaz, M.; Ashby, P.; Lu, Z.; Chu, Y.; Wang, Z.; Ding, X.; Xie, Y.; Chen, J.; Hohman, J. N.; Sanghadasa, M.; Grossman, J. C.; Lin, L. Laser-Sculptured Ultrathin Transition Metal Carbide Layers for Energy Storage and Energy Harvesting Applications. *Nat. Commun.* **2019**, *10* (1), 3112. https://doi.org/10.1038/s41467-019-10999-z.

(5) Yang, Q.; Xu, Z.; Fang, B.; Huang, T.; Cai, S.; Chen, H.; Liu, Y.; Gopalsamy, K.; Gao, W.; Gao, C. MXene/Graphene Hybrid Fibers for High Performance Flexible Supercapacitors. *J. Mater. Chem. A* **2017**, *5* (42), 22113–22119. https://doi.org/10.1039/C7TA07999K.

(6) Yue, Y.; Liu, N.; Ma, Y.; Wang, S.; Liu, W.; Luo, C.; Zhang, H.; Cheng, F.; Rao, J.; Hu, X.; Su, J.; Gao, Y. Highly Self-Healable 3D Microsupercapacitor with MXene–Graphene Composite Aerogel. *ACS Nano* **2018**, *12* (5), 4224–4232. https://doi.org/10.1021/acsnano.7b07528.

(7) Yuan, Y.; Jiang, L.; Li, X.; Zuo, P.; Zhang, X.; Lian, Y.; Ma, Y.; Liang, M.; Zhao, Y.; Qu, L. Ultrafast Shaped Laser Induced Synthesis of MXene Quantum Dots/Graphene for Transparent Supercapacitors. *Adv. Mater.* **2022**, *34* (12), 2110013. https://doi.org/https://doi.org/10.1002/adma.202110013.

(8) Abdolhosseinzadeh, S.; Schneider, R.; Verma, A.; Heier, J.; Nüesch, F.; Zhang, C. (John). Turning Trash into Treasure: Additive Free MXene Sediment Inks for Screen-Printed Micro-Supercapacitors. *Adv. Mater.* **2020**, *32* (17), 2000716. https://doi.org/https://doi.org/10.1002/adma.202000716.

(9) Brousse, K.; Pinaud, S.; Nguyen, S.; Fazzini, P.-F.; Makarem, R.; Josse, C.; Thimont, Y.; Chaudret, B.; Taberna, P.-L.; Respaud, M.; Simon, P. Facile and Scalable Preparation of Ruthenium Oxide-Based Flexible Micro-Supercapacitors. *Adv. Energy Mater.* **2020**, *10* (6), 1903136. https://doi.org/https://doi.org/10.1002/aenm.201903136.

(10) Lin, J.; Peng, Z.; Liu, Y.; Ruiz-Zepeda, F.; Ye, R.; Samuel, E. L. G.; Yacaman, M. J.; Yakobson, B. I.; Tour, J. M. Laser-Induced Porous Graphene Films from Commercial Polymers. *Nat. Commun.* **2014**, *5*, 5714.

(11) Yang, W.; Yang, J.; Byun, J. J.; Moissinac, F. P.; Xu, J.; Haigh, S. J.; Domingos, M.; Bissett, M. A.; Dryfe, R. A. W.; Barg, S. 3D Printing of Freestanding MXene Architectures for Current-Collector-Free Supercapacitors. *Adv. Mater.* **2019**, *31* (37), 1902725. https://doi.org/https://doi.org/10.1002/adma.201902725.

(12) Zhang, W.; Li, R.; Zheng, H.; Bao, J.; Tang, Y.; Zhou, K. Laser-Assisted Printing of

Electrodes Using Metal–Organic Frameworks for Micro-Supercapacitors. *Adv. Funct. Mater.* **2021**, *31* (14), 2009057. https://doi.org/https://doi.org/10.1002/adfm.202009057.
